# A cleavable peptide adapter augments the activity of targeted toxins in combination with the glycosidic endosomal escape enhancer SO1861

**DOI:** 10.1186/s12896-024-00854-5

**Published:** 2024-04-29

**Authors:** Finn J. Schulze, Mazdak Asadian-Birjand, Michael Pradela, Nicole Niesler, Gregor Nagel, Hendrik Fuchs

**Affiliations:** 1 Institute of Diagnostic Laboratory Medicine, Clinical Chemistry and Pathobiochemistry, Charité – Universitätsmedizin Berlin, corporate member of Freie Universität Berlin and Humboldt-Universität zu Berlin, Augustenburger Platz 1, 13353 Berlin, Germany; 2 Interfaculty Institute of Biochemistry, University of Tübingen, Auf der Morgenstelle 34, 72076 Tübingen, Germany

**Keywords:** Molecular adapter, Glycosylated triterpenoid, Endosomal escape, Drug delivery, Cancer treatment, Epidermal growth factor, Dianthin

## Abstract

**Background:**

Treatment with tumor-targeted toxins attempts to overcome the disadvantages of conventional cancer therapies by directing a drug’s cytotoxic effect specifically towards cancer cells. However, success with targeted toxins has been hampered as the constructs commonly remain bound to the outside of the cell or, after receptor-mediated endocytosis, are either transported back to the cell surface or undergo degradation in lysosomes. Hence, solutions to ensure endosomal escape are an urgent need in treatment with targeted toxins. In this work, a molecular adapter that consists of a cell penetrating peptide and two cleavable peptides was inserted into a targeted toxin between the ribosome-inactivating protein dianthin and the epidermal growth factor. Applying cell viability assays, this study examined whether the addition of the adapter further augments the endosomal escape enhancement of the glycosylated triterpenoid SO1861, which has shown up to more than 1000-fold enhancement in the past.

**Results:**

Introducing the peptide adapter into the targeted toxin led to an about 12-fold enhancement in the cytotoxicity on target cells while SO1861 caused a 430-fold increase. However, the combination of adapter and glycosylated triterpenoid resulted in a more than 4300-fold enhancement and in addition to a 51-fold gain in specificity.

**Conclusions:**

Our results demonstrated that the cleavable peptide augments the endosomal escape mediated by glycosylated triterpenoids while maintaining specificity. Thus, the adapter is a promising addition to glycosylated triterpenoids to further increase the efficacy and therapeutic window of targeted toxins.

**Supplementary Information:**

The online version contains supplementary material available at 10.1186/s12896-024-00854-5.

## Background

Specifically targeting cancer cells has been in focus of cancer research over the past decades. Drug effects can be accurately directed by targeting molecular structures that are overly expressed or overly active in cancer cells [1]. Long since, the epidermal growth factor (EGF) receptor (EGFR) has thus been targeted in specific treatments of various cancer types. It is frequently overexpressed in 25–77% of colorectal carcinomas, around 60% of non-small cell lung cancers, 15–30% of breast cancers and more [2–4]. Correspondingly, EGFR-targeted antibodies such as cetuximab and panitumumab as well as EGFR-tyrosine kinase inhibitors such as erlotinib have entered clinical practice. In most cases, however, these drugs have been restricted to patients with metastatic diseases and, while prolonging survival, do not lead to full recovery of patients [5, 6].

Given these shortcomings of unmodified antibodies and small molecules, combining a tumor specific ligand with a toxic moiety is an attempt to raise efficacy of targeted therapies [7]. Toxic moieties in clinical cancer therapy mostly rely on cytostatic agents, radioisotopes or small molecule drugs to exert toxicity [8]. A potential alternative to these commonly used toxic moieties are highly effective protein toxins such as ribosome-inactivating proteins (RIPs). RIPs are a group of mostly plant-derived enzymes, which exert their toxicity by releasing a specific adenine residue of eukaryotic 28S ribosomal RNA (N-glycosidase activity) [9]. Type I RIPs such as dianthin, saporin or gelonin solely consist of a catalytic A-chain, while type II RIPs like ricin or abrin provide an additional cell-binding B-chain [10]. Due to this additional domain, type II RIPs enter cells and exert non-specific cytotoxicity. In contrast, the absence of the B-chain makes type I RIPs promising candidates for the development of targeted toxins [11]. In particular, the type I RIP dianthin is known to have a high potential for successful use in targeted tumor therapies [12–15].

Yet, merely combining a targeting component with a protein toxin has led to unsatisfactory results. Several mechanisms may contribute to the inefficiency of targeted protein toxins. Firstly, they might remain bound to the outside of the cell. If receptor-mediated endocytosis takes place, targeted protein toxins may immediately be recycled back to the cell surface alongside the receptor or transported to lysosomes with subsequent degradation [16].

Considering this, solutions to ensure cytosolic delivery of targeted protein toxins are indispensable.

Growing attention is driven towards certain glycosylated triterpenoids, which are plant-derived amphiphilic glycosides with a triterpenoid backbone [17]. By enhancing endosomal escape, they increased target cell-specific cytotoxicity of RIPs in combination with a targeting ligand up to 4,000,000-fold in vitro and are therefore referred to as endosomal escape enhancers (EEEs) [18]. The EEE effect has repeatedly been shown in vivo [19]. It is not a general characteristic of glycosylated triterpenoids but is limited to a small number of compounds such as SO1861 that is derived from *Saponaria officinalis* L. [19].

**Fig. 1:**
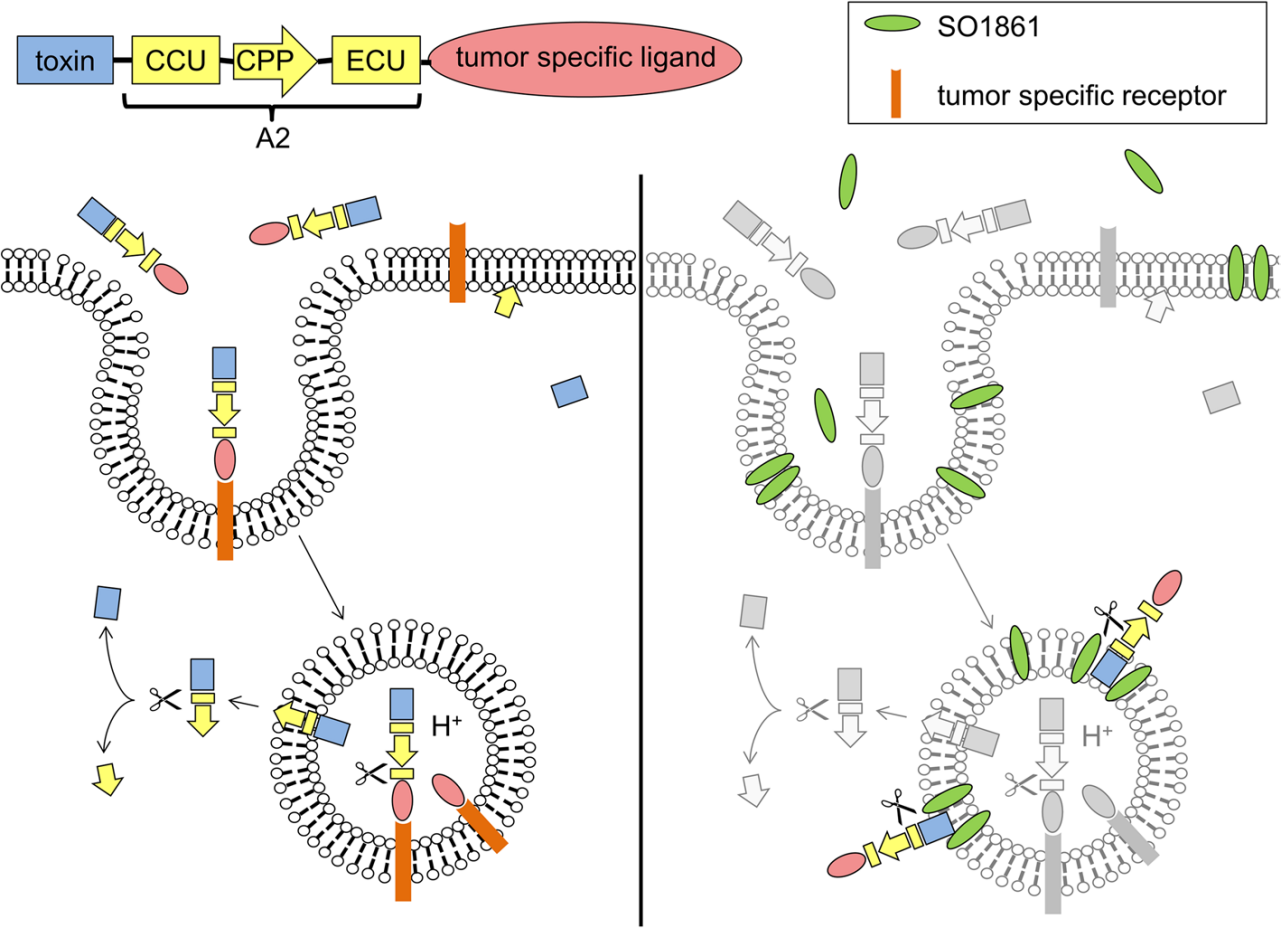
Mechanism of action of the A2 (left-hand side) and model for combination of the A2 and SO1861 (right-hand side). On the left, the figure depicts step-by-step how the CPP and cleavage of the A2 peptides ensure that targeted toxins are delivered to the cytosol. A model of how the combination of the A2 and SO1861 mediates endosomal escape of targeted toxins is depicted on the right. In this part of the image, the effects mediated by the A2 are shown in greyscale, while additional effects through SO1861 are displayed in colour. The CPP is derived from the preS2-domain of the hepatitis B virus surface antigen. While the exact mechanism of action of this peptide is not known, the cell penetrating property can be related to an amphipathic α-helix in its structure [23]. The drawing was created following picture elements from previous publications [19, 24]

In search of other strategies to ensure cytosolic drug delivery, Keller et al. designed a cleavable peptide adapter composed of a *C*-terminal endosomal cleavable unit (ECU, also referred to as ECP [20–22]), an *N*-terminal cytosolic cleavable unit (CCU, also referred to as CCP [20–22]) and a cell penetrating peptide (CPP) in between (Fig. 1) [24]. Due to reduced stability of the original adapter, Heisler et al. modified a cleavage site within the ECU and since then referred to the modified adapter as Ad* or, as in this study, as A2 [20–22]. When inserting the adapter A2 between the RIP saporin and the targeting moiety EGF (Saporin-A2-EGF), an improved anti-cancer effect in mice with EGFR-positive tumors and simultaneously lesser side effects were observed in comparison to Saporin-EGF [22]. As shown on the left of Fig. 1, cytosolic drug delivery by the A2 can be explained by initial cleavage of the ECU in the endosome, which separates the ligand and reveals the CPP. The latter is then no longer blocked and can penetrate the endosomal membrane. After entering the cytosol, the CCU is then cleaved and separates the CPP from the toxin, which forces the toxin to stay in the cytosol where it exhibits its cytotoxicity [20].

Both, the glycosylated triterpenoids and the adapter A2 represent promising candidates for controlled cytosolic drug delivery of targeted toxins in cancer therapy. However, while glycosylated triterpenoids have been broadly investigated in the past, studies examining whether the A2 augments their endosomal escape enhancement were not conducted.

In this study, we investigated the activity of two targeted protein toxins (consisting of dianthin and EGF), one with (DAE) and the other without (DE) the adapter A2, on EGFR-positive cells with and without addition of the glycosylated triterpenoid SO1861. To do so, we generated a new targeted toxin composed of the RIP I dianthin, the A2, and EGF. In addition, a Strep-tag C-terminal of the EGF was introduced to obtain a pure fusion protein (DAES). Most importantly, we examined if further increase of SO1861-mediated endosomal escape can be achieved by inserting the A2 into the targeted toxin.

## Results

### Expression and purification of ^His^Dianthin-EGF (DE), ^His^Dianthin-A2-EGF (DAE) and ^His^Dianthin-A2-EGF^Strep^ (DAES)

**Fig. 2:**
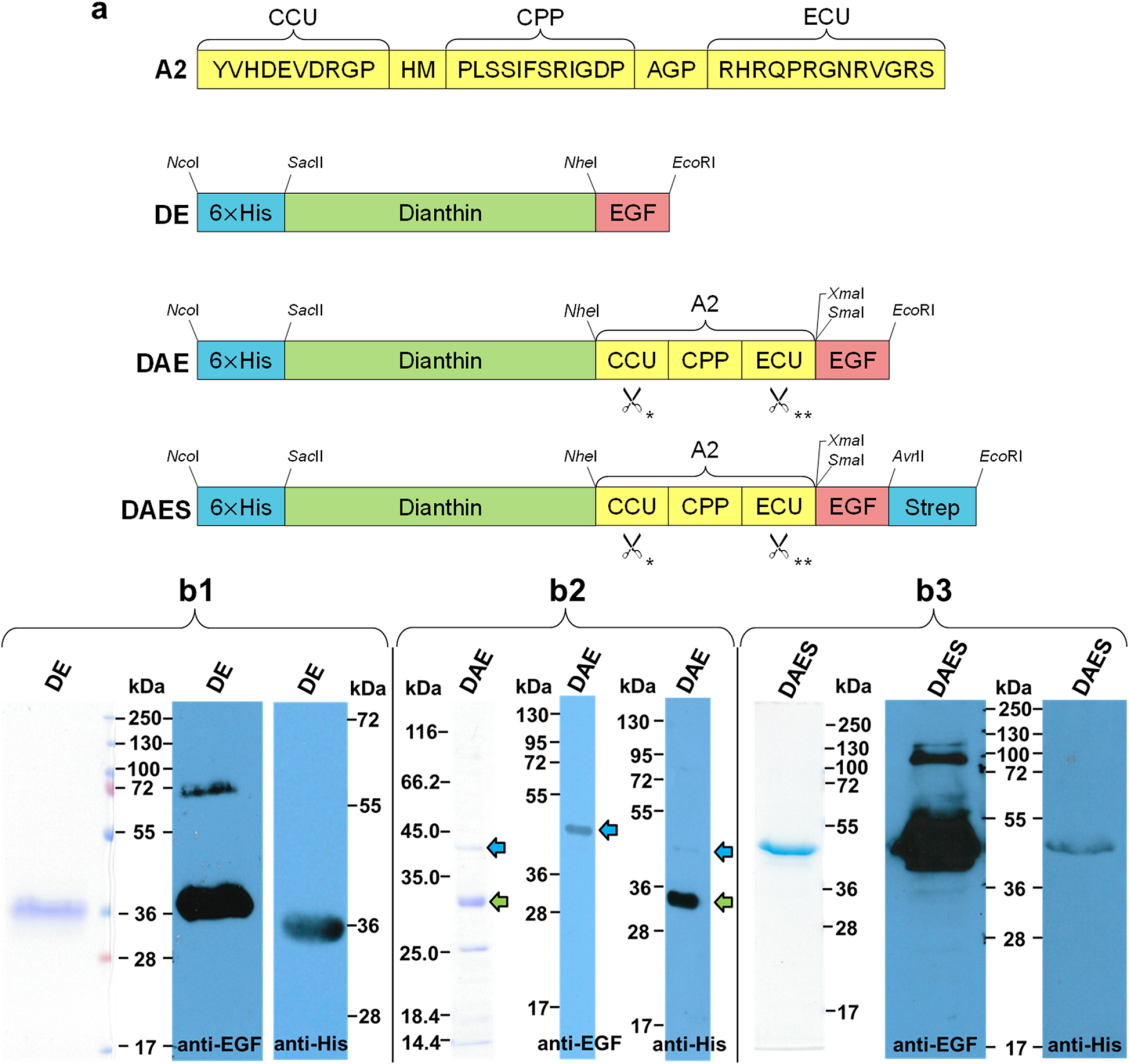
Overview of the different protein structures and results of purification of DE, DAE and DAES. **a** The amino acid sequence of the adapter A2 and the general structures of the three different proteins produced in this study are depicted along with cleavage sites for restriction enzymes. *amino acids YVHD↑EVD↑RG↑P containing cleavage sites for cytosolic proteases including caspase-3 (second arrow in the sequence). **amino acids RHRQPR↑GNRVGR↑S containing cleavage sites for furin (endosomal enzyme). **b** Each purified protein is shown in Coomassie and was detected by an anti-EGF and an anti-His antibody by Western blotting. The gels and blots are cropped. Full-length gels/blots are presented in Additional file 1. (b1) Purified DE is shown. The additional band at about 72 kDa corresponds to a DE-dimer. (b2) Result after purification of DAE by Ni-NTA and chitin column affinity chromatography. The band corresponding to intact DAE is pointed out with a blue arrow, while a large fragment after cleavage is indicated with a green arrow. (b3) Result after purification of DAES. The band between 72 and 100 kDa in the anti-EGF Western blot corresponds to a dimer of the protein

An overview of the different protein structures and the purification results is given in Fig. 2. The molecular mass and the number of base pairs corresponding to each protein are shown in Table 1.

**Table 1:** Molecular mass and number of base pairs of the different proteins

	molecular mass	number of base pairs
DAES	41.9 kDa	1118 bp
DAE	40.7 kDa	1088 bp
DE	36.2 kDa	966 bp
^His^Dianthin	29.7 kDa	794 bp
A2	4.7 kDa	128 bp
EGF	6.3 kDa	163 bp
Strep-tag	1.2 kDa	33 bp
DAES after cleavage by furin	Large fragment: 34.2 kDa Small fragment: 7.7 kDa	
DAES after cleavage by caspase-3	Large fragment: 30.7 kDa Small fragment: 11.2 kDa	

After expression in *E. coli* NiCo21 bacteria, DE was isolated by nickel nitrilotriacetic acid chromatography (Ni-NTA) and chitin column affinity chromatography yielding a pure protein solution.

While the expression of DAE delivered the desired protein, satisfying purity was not reached with Ni-NTA and chitin column affinity chromatography. As shown by Western blotting, DAE was detectable at the expected molecular mass when using an anti-EGF-antibody. However, an additional band with higher intensity was visible at a lower molecular mass after use of an anti-His-antibody. These findings indicated distinct cleavage of the protein throughout the expression and purification process. Cytotoxicity assays and other experiments would not be conclusive with a major part of the protein being cleaved beforehand.

In order to separate the intact protein from any of its fragments, purification relied upon protein tags on both the *N*-terminal and the *C*-terminal end of the protein. For this, an additional Strep-tag was cloned to the *C*-terminal end of the DAE DNA. Following the expression of the modified protein, Ni-NTA affinity chromatography in combination with Strep-Tactin-purification allowed isolation of intact DAES (see also Additional file 2).

### In vitro cleavability of the ECU and CCU of DAES

ECU and CCU were designed by inserting cleavage sites for furin derived from diphtheria toxin and *Pseudomonas* exotoxin and for caspases, respectively [20] (Fig. 2). Thus, in order to prove the cleavability of the ECU and CCU, DAES was incubated for a period of 20 h with furin, which is present in endosomes, and for 1 h with caspase-3, which exists in the cytosol.

**Fig. 3:**
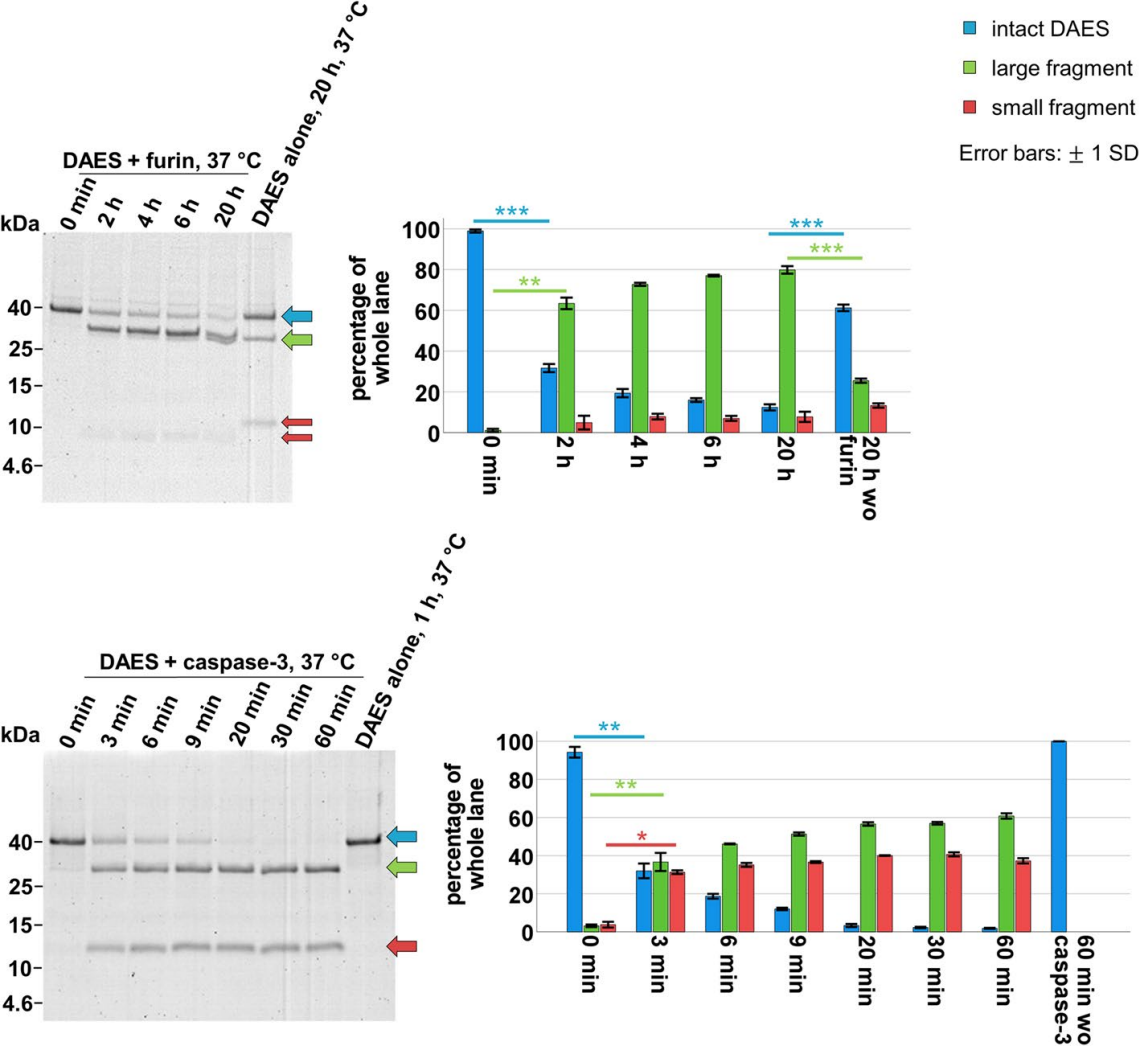
In vitro cleavability of the ECU and CCU of DAES. The gels were stained with Coomassie and analysed with a molecular imager. Cleavage by furin, which is present in the endosome, is shown on the upper half while cleavage by the cytosolic enzyme caspase-3 is depicted below. Cleavage conditions: DAES (3.125 μmol/L) and furin (14.125 U/mL) in appropriate buffer (100 mM HEPES, 2 mM CaCl_2_, 0.2% (v/v) Triton X-114, pH 7.0); DAES (3.75 μmol/L) and caspase-3 (3 μg/mL) in caspase buffer (25 mM HEPES, 10 mM DTT, 0.1% (w/v) CHAPS, pH 7.4). The controls were each incubated in the corresponding buffer. The blue arrow in both images points out the intact DAES protein, while the green arrow shows the *N*-terminal fragment of the cleaved protein and the red arrow points at the *C*-terminal fragment. Decrease of intact DAES and increase of the fragments over time is shown by the graphs on the right. Both assays were repeated in three independent experiments. The most important results of the statistical analysis by Student’s paired t-test (Student’s unpaired t-test for comparison to incubation without enzyme) are included. *P* < 0.05 was considered statistically significant. P < 0.05, *; *P* < 0.01, **; *P* < 0.001, ***. The gels are cropped. Full-length gels are presented in Additional file 3

The entire DAES protein consists of 371 amino acids and has a mass of about 42 kDa. Table 1 shows the molecular mass of the large and small fragments after cleavage of the ECU by furin or of the CCU by caspase-3, respectively. Using sodium dodecyl sulfate-polyacrylamide gel electrophoresis (SDS-PAGE) with 10% acrylamide and 10% glycerol, separation of low molecular mass proteins was achieved and differences between the fragments were visible as shown in Fig. 3 (see also Additional file 3). Following background subtraction, cleavage of DAES over time was assessed by quantification of band intensities (see also Additional file 4).

In both assays, SDS-PAGE revealed a pure solution of intact DAES immediately after addition of furin or caspase-3. A clear decrease of intact DAES and increase of the large cleavage fragment was visible after 3 min incubation with caspase-3 and 2 h with furin. For the caspase-3 assay, also the small cleavage fragment obviously increased within the first 3 min. With both enzymes, samples taken after longer incubation time demonstrated a continuous decrease of intact DAES and increase of the large cleavage fragment. Without addition of the enzyme, incubation of DAES over 1 h in caspase-3 buffer did not lead to any measurable cleavage. DAES incubation for 20 h in enzyme-free furin buffer showed some cleavage but evidently less cleavage than the corresponding sample containing the enzyme. Addition of furin revealed a small cleavage fragment at about the expected molecular mass, but definite increase could not be observed. Notably, the small cleavage fragment resulting from enzyme-free incubation of DAES in furin buffer had a slightly higher molecular mass than the small fragment after furin cleavage, indicating cleavage at a different site.

**Fig. 4:**
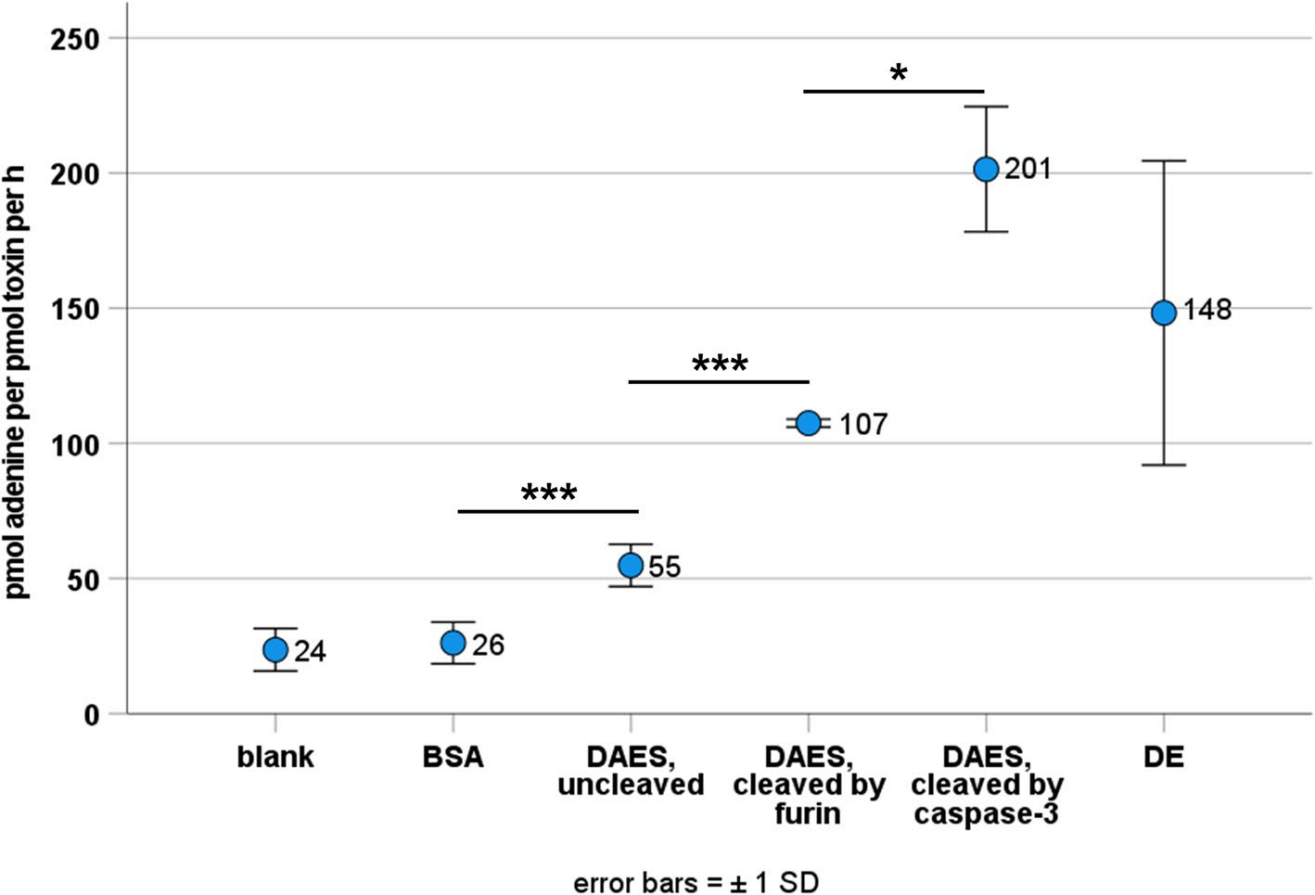
Enzymatic activity of the different proteins measured by adenine release assay. The adenine release is expressed as pmol adenine per pmol toxin per hour. All data points result from at least three replicates. Statistical significance was determined by Student’s unpaired t-test. P < 0.05 was considered statistically significant. P < 0.05, *; P < 0.01, **; P < 0.001, ***. There was no statistical significance between blank and BSA as expected and between DE and cleaved DAES, whether by caspase-3 or furin. All other differences were statistically significant

Enzymatic activity of DE, DAE and of DAES before and after cleavage was determined using adenine release assays (Fig. 4, see also Additional file 5). Cleavage of the adapter restores the enzymatic activity that is partly reduced in non-cleaved DAES. The adenine release mediated by the partly cleaved DAE was only measured once and determined to be 113 pmol∙pmol^−1^∙h^−1^.

### Cytotoxicity assays

Using MTT (3-[4,5-dimethylthiazol-2-yl]-2,5 diphenyl tetrazolium bromide) assays, cytotoxicity of DE and DAES with or without SO1861 (125 ng/mL) was determined on the EGFR-positive colon cancer cell line HCT 116 and the mammary gland carcinoma cell line MDA-MB-453, which expresses only low amounts of EGFR [25, 26] (see also Additional file 6).

**Fig. 5:**
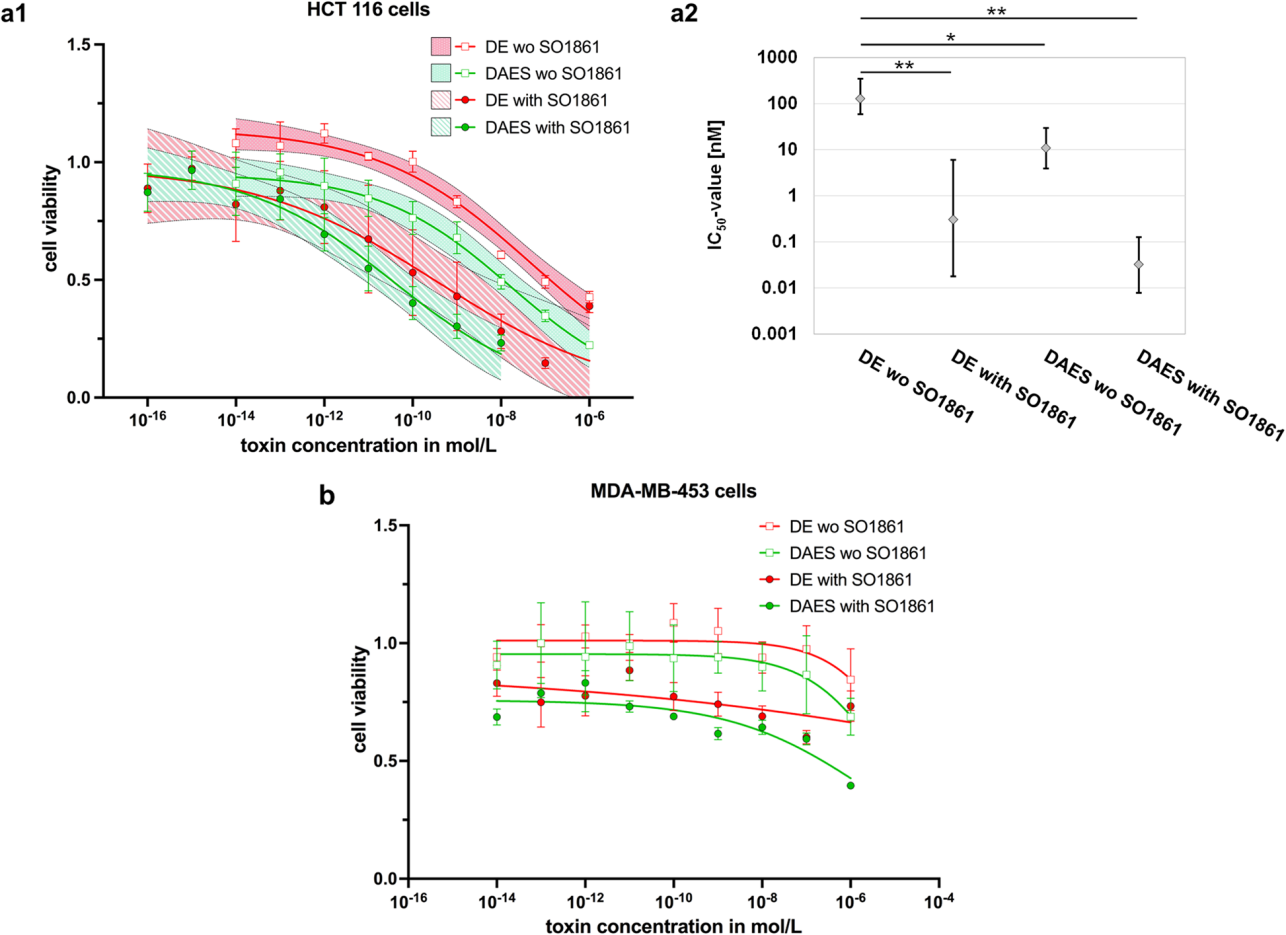
Cytotoxicity assays of DE and DAES with or without SO1861. Cytotoxicity of DE and DAES was evaluated by incubation of HCT 116 (EGFR-positive) and MDA-MB-453 (low EGFR-expression) cells with different concentrations of the toxins ranging from 0.1 pM to 1 μM, with or without addition of SO1861 at a concentration of 125 μg/mL. After 48 h incubation, the viability of the cells was measured by MTT assay. **a1** The curves result from four parameter logistic regression by GraphPad Prism. The 95% confidence interval for each curve is indicated. The displayed data points each represent the mean ± SEM of three or four independent assays each performed in triplicate. As an exception, the two data points of DE at a concentration of 100 nM and 1 μM with addition of SO1861 rely on only two experiments. **a2** Overview over IC_50_-values for HCT 116 cells, including 95% confidence interval and statistical significance. A logarithmic scale is used for the y-axis. Statistical significance was assessed by Student’s unpaired t-test. P < 0.05 was required for statistical significance. P < 0.05, *; P < 0.01, **; P < 0.001, ***. **b** The curves result from four parameter logistic regression by GraphPad Prism. The 95% confidence intervals could not be calculated because the sigmoid curves did not reach the baseline level due to the low toxicity for MDA-MB-453 cells. The displayed data points each represent the mean ± SEM of three or four independent assays each performed in triplicate. Additional graphs that allow ideal comparison of the effect on HCT 116 cells with the effect on MDA-MB-453 cells can be found in Additional file 8

Both DE and DAES clearly showed dose-dependent toxicity on HCT 116 and MDA-MB-453 cells (Fig. 5). The inhibitory concentrations at 50% cell survival (IC_50_ values, required dose of the targeted toxins yielding 50% cell survival) and toxicity enhancement factors are shown in Additional Tables 5 and 6 of Additional file 7. Receptor target indices and values for the gain in specificity are shown in Table 7 of Additional file 7. Due to missing cytotoxicity as expected for off-target cells, the IC_50_-value for the combination of DE and SO1861 for treatment of MDA-MB-453 cells could not be determined by four parameter logistic regression.

Each factor of enhancement is calculated by division of the corresponding IC_50_-values and illustrates, to what extent one treatment is superior to another regarding its intended cytotoxicity. SO1861 evidently enhanced the cytotoxicity of both toxins towards EGFR-positive HCT 116 cells. It exhibited a 430-fold enhancement for DE and a 370-fold enhancement for DAES. Given that SO1861 was used at a non-toxic concentration, this observation suggests a synergistic effect. Addition of the A2 enhanced cytotoxicity by a factor of twelve in the absence of SO1861 and by an additional factor of ten in its presence. Notably, the combination of the A2 and SO1861 resulted in an enhancement factor of 4300 when comparing it to the use of DE alone. These findings indicated superiority of the combined use of endosomal escape enhancers with different molecular mechanisms regarding cytotoxicity towards target cells.

Looking at MDA-MB-453 cells, cell viability never exceeded 83% when SO1861 was added even at lowest toxin concentrations. This indicated toxicity of SO1861 itself towards these cells. To assess, whether use of SO1861 and/or the A2 decreased or increased specificity, we calculated receptor targeted indices by dividing the corresponding IC_50_-values for HCT 116 cells and MDA-MB-453 cells. Gain or loss in receptor specificity was then calculated by division of receptor target indices. Despite some toxicity of SO1861 towards MDA-MB-453 cells, addition of SO1861 led to a 15-fold gain in receptor specificity for DAES. Importantly, DAES without use of SO1861 showed to be 3.5-times more specific than DE, which demonstrated increase of specificity through the A2. In comparison to single treatment with DE, DAES together with SO1861 displayed a 51-fold gain in specificity. Although MTT assays for MDA-MB-453 cells were at least performed in triplicate, the 95% confidence interval of the IC_50_-value could not be calculated due to a lack of toxicity on these off-target cells at the tested concentration range. Higher concentrations of DE and DAES were not feasible due to limited yield and solubility of the proteins.

IC_50_-values of the cytotoxicity assays with HCT 116 cells were examined in terms of statistical significance (*p* < 0.05) by Student’s unpaired t-test. Although the IC_50_-value of DAES with SO1861 was low at 0.03 nM while the corresponding value for the combination of DE with SO1861 was found to be ten times higher (0.3 nM), the difference between these therapies did not reach statistical significance. On the other hand, both DE with SO1861 (*p* = 0.002) and DAES without SO1861 (*p* = 0.011) significantly surpassed DE alone. And importantly, DAES in combination with SO1861 was as well significantly superior to DE without SO1861 (*p* = 0.002). All other constellations did not show significant differences. Figure 5 includes an overview of the different IC_50_-values, 95% confidence intervals and significance. The relatively large differences between the individual experiments can be explained by slight differences in the sensitivity of the cells between the individual batches and minimal spreading in the concentration of SO1861. However, the individual experiments are consistent in themselves (Additional file 6).

### Plasma stability of DE and DAES

**Fig. 6:**
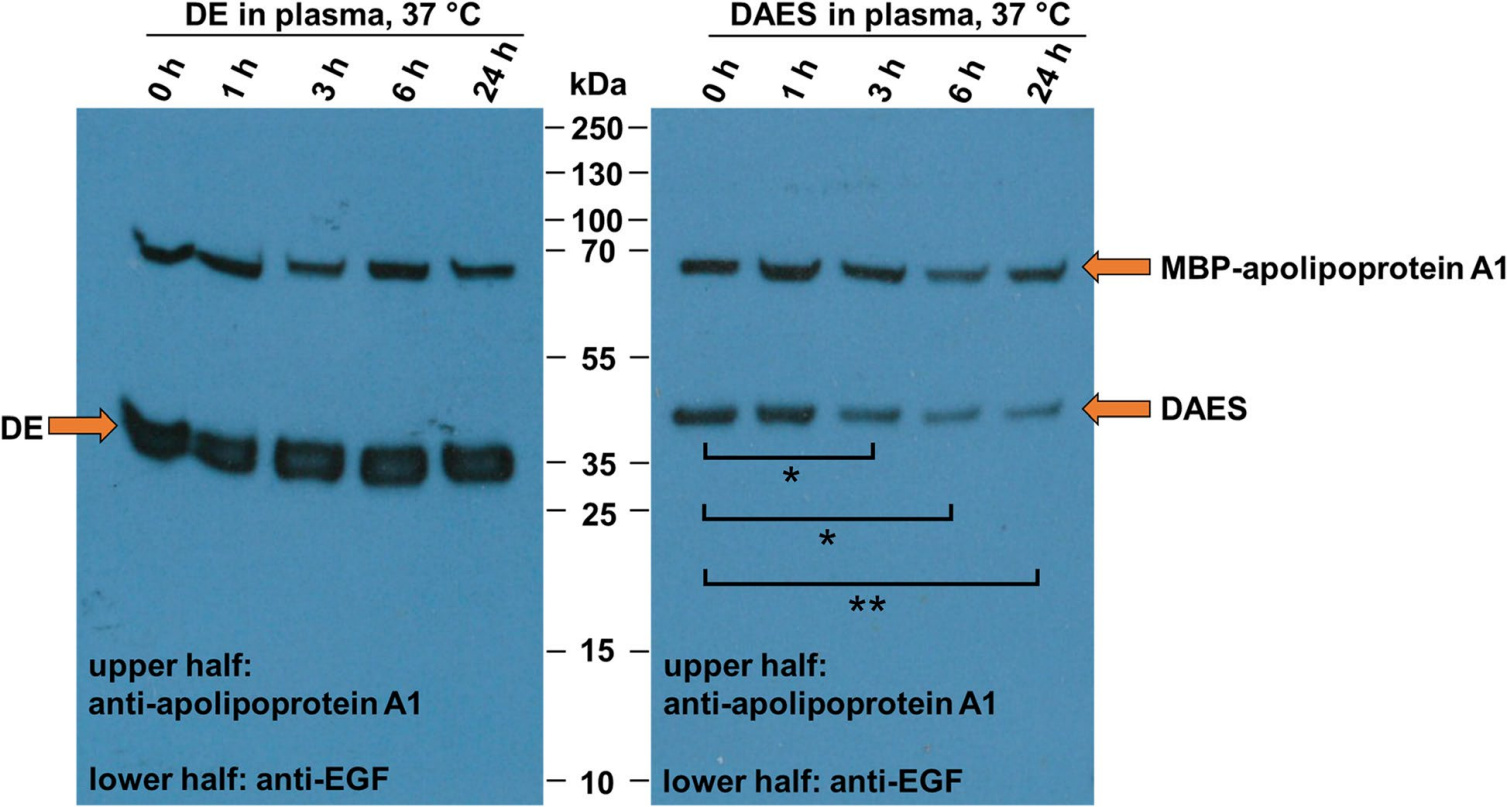
Plasma stability of DE and DAES. Samples of DE- and DAES-incubation in human plasma for different time intervals were analysed by Western blotting. For quantification, the DE and DAES bands were related to the band intensity of spiked MBP-apolipoprotein A1. Statistically significant results as determined using the Student’s paired t-test are indicated. *P* < 0.05 was required for statistical significance. *P* < 0.05, *; *P* < 0.01, **; *P* < 0.001, ***. The blots are cropped. Full-length blots are presented in Additional file 9

To assess plasma stability of DE and DAES, both proteins were incubated in human plasma at 37 °C for 24 h. Samples were taken at different time intervals and an identical amount of His-tagged apolipoprotein A1, fused to maltose-binding protein (MBP), was added to each sample. After the proteins were recaptured by Ni-NTA, they were analysed by Western blot with anti-EGF and anti-apolipoprotein A1 antibodies (Fig. 6, see also Additional file 9). Intensity of the bands corresponding to DE (at about 36 kDa) or DAES (at about 42 kDa) was quantified and then normalized by dividing through the band intensity of MBP-apolipoprotein A1 (MBP-apolipoprotein A1 69 kDa) (see also Additional file 10).

Incubation of DE in human plasma revealed high protein stability. There was no statistically significant difference between the initial band intensity before incubation and the band intensities after 1, 3, 6 or 24 h. In fact, the mean intensity of the DE band in the 24 h lane was at about 122% (SD 34%) of the corresponding band in the 0 h lane, making a substantial loss of protein unlikely.

Stability was analogously investigated for DAES. The proportion of non-cleaved protein was clearly reduced within the period studied. In relation to the initial band intensity before incubation, 59% (SD 17%) of this intensity remained after 3 h. Nevertheless, the DAES band of the 24-h-sample still made up about 47% (SD 8%) of the original intensity. Thus, a considerable amount of intact protein was preserved throughout the entire incubation period. The band intensities of DAES after 3 h (*p* = 0.027), 6 h (*p* = 0.013) and 24 h (*p* = 0.004) were significantly lower than the initial band intensity before incubation.

In order to detect low molecular mass fragments of DAES with the anti-EGF antibody, the Western blot membranes had been incubated in 0.5% glutaraldehyde before blocking. However, no band at the expected low molecular mass was visible in any of the replicates.

## Discussion

Up to date, several different RIPs have been assessed as targeted drugs for cancer therapy. In contrast to dianthin, targeted toxins with other RIPs have already been evaluated in clinical trials [27, 28]. Yet, none of the clinical trials with ricin, gelonin or saporin up to now resulted in market entry. Similarly, very few targeted protein toxins other than RIPs have been in clinical use. Rare exceptions such as denileukin diftitox (containing truncated diphtheria toxin) or moxetumomab pasudotox (containing truncated *Pseudomonas* exotoxin) suffer from low specificity and a narrow therapeutic window [29, 30].

Therefore, efficacy of targeted protein toxins urgently needs to be improved, for example by making use of the adapter A2 or co-treatment with glycosylated triterpenoids, both of which present different and thereby potentially complementary endosomal escape mechanisms.

Up to date, the mechanism of action of the triterpenoid-mediated enhancement is not yet clear. It has been proven that endosomal escape by these EEEs depends on the pH-shift from 6.5 in the early endosome to 4.5 in the endolysosome. It could therefore be possible that glycosylated triterpenoids interact with the corresponding protein in a pH-dependent manner. The endosomal escape itself might then be mediated by interactions between the EEEs, cholesterol and other components of the vesicle’s membrane [19]. Here, we observed toxicity of SO1861 on the EGFR-negative cell line MDA-MB-453 at a concentration of 125 ng/mL. While toxicity of SO1861 has been reported earlier, the toxic effect of this EEE had until now only been registered at concentrations of at least 500 ng/mL [14]. Hence, we consider toxicity at 125 ng/mL to be restricted to this specific cell line and the given culture conditions. Apart from that, the addition of SO1861 to DAES nonetheless led to a gain in specificity. Despite this, it might be beneficial for future research to connect SO1861 to a targeting moiety. In previous work, the in vivo efficacy of tumor-targeted RIPs was enhanced by addition of other types of endosomal escape agents that were bound to tumor-specific antibodies [31, 32].

A crucial challenge of this study was to achieve a balance between sufficient cleavability of the A2 to enhance cytosolic delivery on the one hand and satisfactory stability for circulation to the site of action on the other hand. We added a *C*-terminal Strep-tag to His-tagged DAE enabling us to successfully express and purify a targeted toxin containing a molecular, cleavable peptide adapter. Without the additional *C*-terminal tag, processing of both DAE in this study and the protein ^His^Saporin-A2-EGF in a former study by Fuchs et al. resulted in lower purity [22].

Adenine release assays revealed lower enzymatic activity of DAES in comparison to DE. However, adenine release significantly increased after cleavage by furin and was even higher after caspase-3 cleavage. Hence, the functionality of the construct is not diminished. Endosomal and cytosolic enzymes will cleave the ECU and CCU and remove the Strep-tag, EGF and most of the A2. Additionally, functionality of DAES is supported by the MTT assays. Indeed, reduced activity of dianthin in non-cleaved DAES will diminish unspecific side effects comparable to activable pro-drug therapies.

Cleavability of the ECU and the CCU was proven by enzymatic assays with furin and caspase-3 through obvious decrease of DAES and increase of the cleavage products. The low increase of the small cleavage fragment during incubation with furin is presumably due to insufficient detection. Notably, cleavage by furin needed a much longer incubation time than cleavage by caspase-3. In fact, during development of the adapter, the cleavage sites within the ECU had been modified towards less optimal cleavage sites to achieve greater stability [20]. Although furin has a broad pH optimum between 5 and 8, cleavage might be accelerated due to pH decrease during transport to late endosomes [33].

A cleavable adapter comprising a CPP may theoretically bring dangers. Looking at the lack of cytotoxicity of DAES towards non-target cells with a low degree of EGFR-expression, we consider substantial cell penetration without cleavage of the ECU to be very unlikely. Yet, if cleavage of the ECU occurs outside of cancer cells, the CPP is exposed and allows the penetration of any cell. However, the ECU of DAES appeared to be quite stable in our furin assays. Moreover, in vivo experiments with ^His^Saporin-A2-EGF did not only reveal superiority over ^His^Saporin-EGF in terms of tumor growth inhibition. Rather, the use of the A2 in treatment of tumor-bearing mice even resulted in lesser side effects, allowing for repeated administration [22].

When assessing stability of the proteins in human plasma, DAES clearly showed to be less stable than DE. However, as almost half of the original band intensity remained after 24 h, we expect a pharmacological effect in vivo similar to that observed with ^His^Saporin-A2-EGF [22].

With all this in mind, the A2 on the one hand appears to be both stable and cleavable enough to exert target-specific toxicity in vitro which might translate to in vivo settings. On the other hand, it might simultaneously be cleavable and thus unstable enough to reduce side effects in living organisms due to continuous removal from the circulation.

Considering the cytotoxicity assays reported in the present work, the activity of both chimeric toxins on cancer cells correlates with the degree of EGFR expression confirming previous work of Bachran et al. that describes a clear correlation of EGFR expression to the sensitivity towards an EGFR-targeted RIP [34]. In contrast, in that work, the non-targeted RIP (without EGF) only exhibited a very low toxicity, which was independent of EGFR expression indicating the targeting effect of EGF in EGF-RIP conjugates. While there is sufficient data on the role of EGFR expression, the impact of the internalization rate on the activity of EGFR-targeted RIPs remains unclear. For targets with low internalization rate, optimization of endosomal escape may prove important.

Using EGF in a targeted toxin might potentially induce cell proliferation. In our studies with EGFR-positive HCT 116 cells, low concentrations of DE without addition of SO1861 indeed resulted in cell viability values above 100%. However, the cytotoxic effect clearly predominated when the toxin concentration was increased or when either SO1861 or the adapter A2 or both were added. Several publications with in vitro and in vivo data confirm that the possible proliferative impact is easily outweighed by cytotoxicity when a sufficient toxin dose is used [12, 35, 36].

**Fig. 7:**
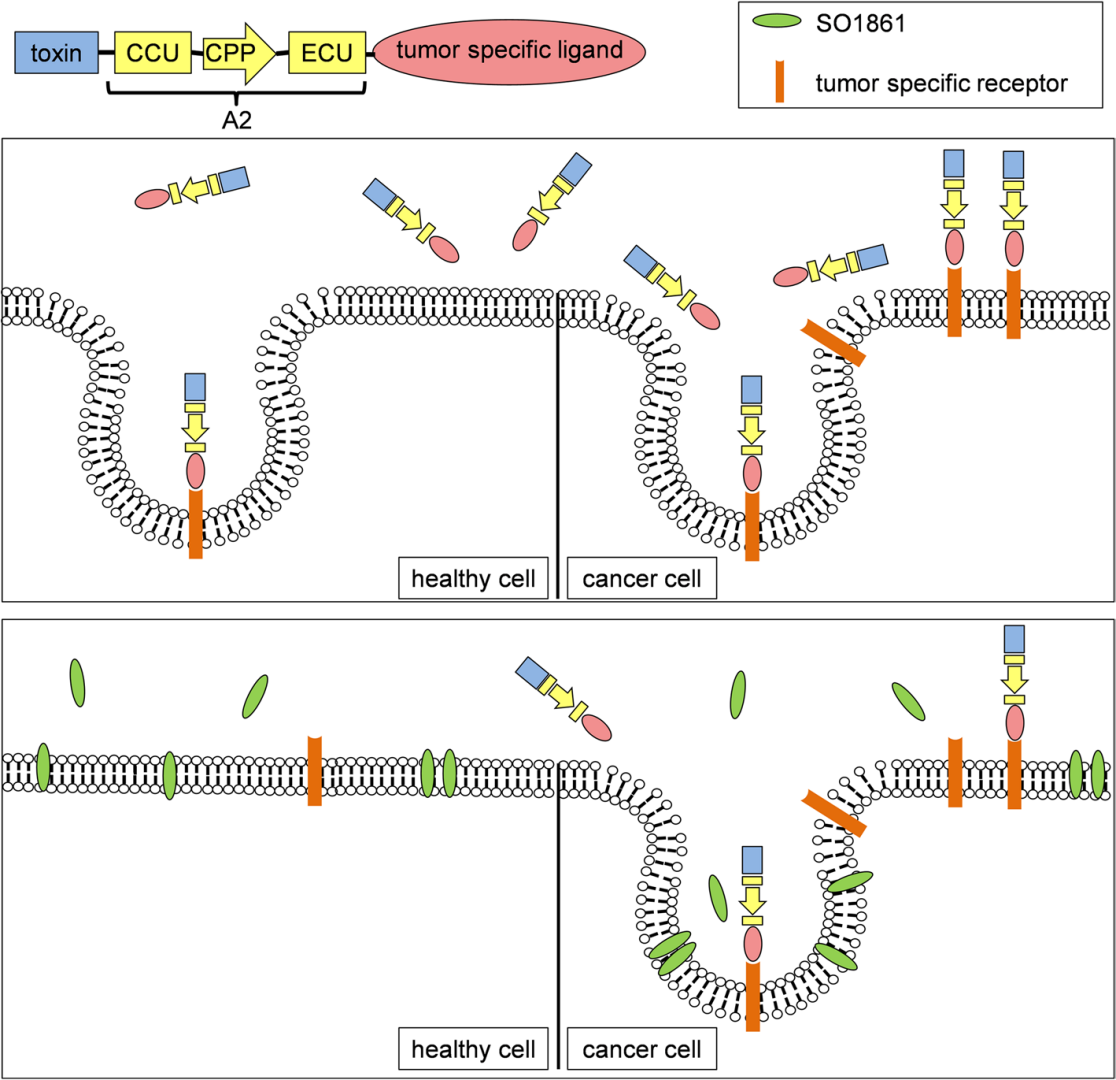
Combination of A2 and SO1861 allows dose reduction. The upper half of the schematic image shows the use of DAES alone, while the lower half depicts the combination of SO1861 and the adapter-consisting construct DAES. The combination allows substantial dose reduction of the targeted toxin, resulting in much fewer toxin molecules binding to healthy cells and thus reducing undesired toxicity. Simultaneously, the toxic effect towards cells that express high levels of EGFR is maintained since SO1861 and the adapter mediate endosomal escape

While the effect of the A2 and especially the endosomal escape enhancement of glycosylated triterpenoids have been assessed in detail in the past, it was unclear whether the combination of a CPP and an EEE would be superior to sole use of an EEE. The cytotoxicity assays carried out to study this issue clearly showed dose-dependent toxicity of DE and DAES on target cells with and without addition of SO1861. To unambiguously assign the observed toxicity to dianthin, we ruled out crucial effects of the cell medium and SO1861 using negative controls. Considering the IC_50_-values of 30 pM (DAES) on the one hand and 300 pM (DE) on the other, there is a clear tenfold improvement of the SO1861-mediated endosomal escape through addition of the A2. This will potentially translate into relatively low toxin doses for in vivo experiments and increases the therapeutic window for targeted toxins (Fig. 7). Apart from that, the adapter also improves efficiency of targeted toxins in the absence of EEEs. This aspect might prove beneficial for in vivo experiments as colocalization of the targeted toxin and the EEEs in vivo is much more challenging than in vitro [37]. With this in mind, in vivo experiments with DAES and SO1861 will be essential for further evaluation of this therapeutic approach.

## Conclusions

This study developed a new targeted toxin consisting of a cleavable, yet sufficiently stable molecular adapter, which can easily be produced and purified by relying on a *C*- and an *N*-terminal tag. Cytotoxicity assays demonstrated that use of the adapter A2 within targeted toxins augments the endosomal escape enhancement by EEEs while maintaining specificity and increasing the therapeutic window. Therefore, the given adapter appears as a promising optimization to endosomal escape of targeted toxins in cancer therapy.

## Methods

### Constructing recombinant plasmids for DAE and DAES

Plasmid DNA of pET11d-^His^Saporin-A2-EGF [20, 21] and pET11d-DE [18] was used in order to construct a pET11d-DAE plasmid. Restriction enzyme digestion of both plasmids with *Nhe*I and *Eco*RI resulted in a pET11d-^His^Dianthin vector on the one hand and an A2-EGF insert on the other (Fig. 2a). After ligation, the pET11d-DAE plasmid was transformed into the chemically competent *Escherichia coli (E. coli)* strain DH5α (New England BioLabs GmbH, Frankfurt am Main, Germany). In order to add a Strep-tag II that consists of eight amino acids to the *C*-terminal end of the DAE protein, polymerase chain reaction was performed on the pET11d-DAE plasmid using the forward primer 5′-TAT ACC ATG GGA CAT CAT CAT CAT C-3′, binding to the 5′ end of the DAE gene, and the reverse primer 5′-A TAT GAA TTC TTA TTT TTC GAA CTG CGG GTG GCT CCA CCT AGG GCG CAG TTC CCA CCA CTT CAG-3′, coding for the Strep-tag II (Metabion, Planegg, Germany). After *Nco*I/*Eco*RI restriction enzyme digestion, the resulting DAES fragment was inserted into an empty pET11d-plasmid. This step was followed by transformation into *E. coli* DH5α. The correctness of both plasmids was assessed by restriction enzyme digestion and by DNA sequence analysis (Eurofins Genomics, Ebersberg, Germany). Each construct used in this study is listed in Table 1 including the number of base pairs and the expected molecular mass.

### Protein expression and purification

The pET11d-DE [18] and pET11d-DAE plasmids were transformed into chemically competent *E. coli* NiCo21 (DE3) (New England BioLabs GmbH, Frankfurt am Main, Germany). After pre-incubation, bacteria were added to 1 L of lysogeny broth medium (LB medium) with 50 μg/mL ampicillin to an optical density (OD) of 0.2 at a wavelength of 600 nm (A600). At 37 °C and 200 rpm, cells were subsequently grown to an OD (A600) of 0.6 to 0.8. Addition of isopropyl β-D-thiogalactopyranoside (IPTG) at a final concentration of 1 mM then induced protein expression. Bacteria were further cultivated for 3 h at 37 °C and 200 rpm. Following centrifugation (20 min, 6000 g, 4 °C), cells were resuspended in 20 mL phosphate buffered saline (PBS, 150 mM NaCl, 8.33 mM Na_2_HPO_4_·2H_2_O, 1.67 mM KH_2_PO_4_, pH 7.4) and frozen at −20 °C. After thawing and addition of a protease inhibitor cocktail (cOmplete, EDTA-free protease inhibitor cocktail, Roche) bacteria were lysed using a French pressure cell (3 cycles, 1500 psi each) (Thermo Fisher Scientific, Waltham, MA, USA). The lysate was then centrifuged (30 min, 30,000 g, 4 °C) and imidazole was added to the supernatant to a final concentration of 20 mM. The *N*-terminal His-tag of the proteins was then used for purification by Ni-NTA chromatography (Protino Ni-NTA Agarose; Macherey-Nagel, Düren, Germany). Samples were eluted with increasing imidazole concentrations (20–250 mM). The fractions containing the desired protein were determined by SDS-PAGE (12%), pooled and afterwards dialyzed against 4 L chitin binding domain buffer (20 mM tris (hydroxymethyl)-aminomethane·HCl, 500 mM NaCl, 1 mM EDTA, 0.1% Tween-20, pH 8.0) at 4 °C. To eliminate bacterial proteins with binding affinity to Ni-NTA agarose, chitin column affinity chromatography was carried out as a further purification step. Again, fractions were analysed with SDS-PAGE. This was followed by dialysis of the pooled fractions containing DE or DAE against 4 L PBS at 4 °C. In a next step, proteins were concentrated with Amicon centrifugal filter devices (10 kDa) (Merck KGaA, Darmstadt, Germany) and their concentration was measured using a bicinchoninic acid assay (Pierce, BCA Protein Assay Kit, Thermo Fisher Scientific, Waltham, MA, USA). The identity of the proteins was verified by SDS-PAGE, Coomassie staining and Western blot either using a mouse monoclonal Penta-His-antibody (Qiagen, Venlo, Netherlands) or using a rabbit polyclonal EGF-antibody (Abcam, Cambridge, United Kingdom).

Expression and purification of DAES were partly performed differently. The main culture was increased to 3 L. IPTG was added earlier at an OD (A600) of 0.5. After centrifugation, bacteria were resuspended in 80 mL PBS and frozen at −20 °C in aliquots. Whenever the protein was needed, only a part of the expression sample was lysed and purified. The extracted protein was then used within the same week. Following purification by Ni-NTA affinity chromatography, the fractions containing DAES were applied to a Strep-tag II specific Strep-Tactin (IBA Lifesciences, Göttingen, Germany) affinity column without prior dialysis and eluted with 2.5 mM desthiobiotin in PBS (pH 8.0). After SDS-PAGE analysis, the corresponding fractions were pooled. Neither dialysis nor concentration was carried out.

The N-glycosidase activity of DE, DAE and DAES was determined by an adenine release assay, which measures the amount of adenine residues released from herring sperm DNA [38]. The assay is described in detail elsewhere [18]. Except for DAE that was only measured once, adenine release assays were performed at least in triplicate for every protein.

### In vitro cleavability of the ECU and CCU of DAES

Functionality of the ECU and CCU was ensured by incubating DAES with either recombinant caspase-3 (Recombinant Human Caspase-3; Bio-Techne GmbH, Minneapolis, MN, USA) or recombinant furin (Furin; New England BioLabs GmbH, Frankfurt am Main, Germany) at 37 °C. For cleavage by caspase-3, DAES with a final concentration of 3.75 μmol/L was incubated with caspase-3 (final concentration 3 μg/mL) in caspase buffer (25 mM HEPES, 10 mM DTT, 0.1% (w/v) CHAPS, pH 7.4). DAES (3.125 μmol/L) was cleaved by furin (final concentration 14.125 U/mL) in an appropriate buffer (100 mM HEPES, 2 mM CaCl_2_, 0,2% (v/v) Triton X-114, pH 7.0). Samples were taken at regular time intervals with a maximum of 1 h for caspase-3 and 20 h for furin. A DAES sample without any added enzyme, but with the corresponding buffer, served as control. All samples were analysed by SDS-PAGE and Coomassie staining. Band intensities were quantified using a molecular imager (VersaDoc, Bio-Rad, Hercules, CA, USA). Both assays were repeated in three independent experiments. Samples of DAES after 1 h incubation with caspase-3 and DAES after 20 h incubation with furin were additionally used for adenine release assays as described above.

### Cell culture and cytotoxicity assays

The human cell line HCT 116 (ATCC, Manassas, VA, USA) is one of three cell lines that were isolated from a colon cancer patient and is shown to overexpress EGFR [39]. Here, HCT 116 cells were cultured in McCoy’s medium (Gibco, Thermo Fisher Scientific, Waltham, MA, USA). MDA-MB-453 cells (ATCC, Manassas, VA, USA) in contrast express low amounts of EGFR [25, 26]. This cell line has been isolated from the metastatic site of a patient with mammary gland carcinoma [40]. In this study, these cells were cultured in Dulbecco’s modified eagle medium (DMEM) (Gibco, Thermo Fisher Scientific, Waltham, MA, USA). Both media were supplemented with 10% fetal bovine serum (BioChrom KG, Berlin, Germany), 100 U/mL penicillin as well as 100 μg/mL streptomycin (Merck KGaA, Darmstadt, Germany). Cells were grown at 37 °C and 5% CO_2_.

To examine, whether HCT 116 and MDA-MB-453 cells express EGFR, cells were lysed and analysed as described in previous work [41]. Western blot was conducted with a rabbit monoclonal EGFR-antibody (Abcam, Cambridge, United Kingdom) and a mouse monoclonal β-Actin-antibody (Merck KGaA, Darmstadt, Germany) as loading control (see also Additional file 11).

For cytotoxicity assays, SO1861 was isolated from the roots of *Saponaria officinalis* L. as described elsewhere [42]. The highest non-toxic concentration of SO1861 was determined for HCT 116 cells. The same concentration was then as well applied to the MDA-MB-453 cell line. Cells were cultivated in 96-well plates (HCT 116 cells: 3000 cells/well, MDA-MB-453 cells: 30,000 cells/well). 24 h after seeding the cells, DE or DAES (final concentrations from 1 μM to 10 fM for both toxins) were added with or without additional SO1861 (final concentration: 125 ng/mL). Cell viability was assessed 48 h later by MTT assay [43]. Cell viability values were calculated by dividing the mean absorption for each condition by the mean absorption of a control (neither toxin nor SO1861 added).

Additional MTT assays were performed with the mouse fibroblast cell lines NIH3T3 and HER14 (NIH3T3 cells stably transfected with human EGFR) [41]. The cells were cultured in DMEM supplemented with 10% fetal bovine serum, 100 U/mL penicillin as well as 100 μg/mL streptomycin and were grown at 37 °C and 5% CO_2_. The results of these MTT assays are reported in Additional file 12.

### Plasma stability of DE and DAES

To assess the stability of DE and DAES, proteins were incubated in 66% human plasma at 37 °C. Samples were taken out of both reaction batches at certain time intervals within a maximum of 24 h. The targeted toxins were recaptured from most of the plasma components using Ni NTA Agarose. In advance, His-tagged apolipoprotein A1 fused to MBP was spiked to each sample and served as a reference substance for quantification. After purification, cleavage of the proteins was analysed by SDS-PAGE and Western blot. To achieve fixation of low molecular mass proteins that might result from cleavage of DAES, the nitrocellulose membranes were treated with 0.5% glutaraldehyde before blocking. While DE and DAES were detected using the anti-EGF-antibody as described above, MBP-apolipoprotein A1 was detected by a separate mouse monoclonal antibody against apolipoprotein A1 (Thermo Fisher Scientific, Waltham, MA, USA). This assay was repeated in three independent experiments.

### Statistics

Cytotoxicity assays were analysed with GraphPad Prism 9 (GraphPad Software, San Diego, CA, USA). Here, four parameter logistic regressions were performed. The value for cell viability at infinite concentration was set to zero. The resulting curve fits were used to calculate the required dose of the targeted toxins yielding 50% cell survival (inhibitory concentration at 50% cell survival, IC_50_ values). Calculation of the IC_50_-values included the results of at least three replicates. Statistical significance of the IC_50_ values was determined by Student’s unpaired t-test.

Student’s unpaired t-test was as well used to evaluate the statistical significance of adenine release assays. For statistical analysis of the cleavage by caspase-3 or furin as well as the protein stability in human plasma, Student’s paired t-test was used.

A *p*-value below 0.05 was required for confirming significance.

### Supplementary Information

**Supplementary Material 1.**

## Data Availability

All data generated or analysed during this study are included in this published article and its additional files.

## Abbreviations

A2: Molecular cleavable peptide adapter consisting of CCU, CPP and modified ECU
ADC: Antibody-drug conjugate
BCA: Bicinchoninic acid
BSA: Bovine serum albumin
CCU: Cytosolic cleavable unit
CPP: Cell penetrating peptide
DAE: ^His^Dianthin-A2-EGF
DAES: ^His^Dianthin-A2-EGF^Strep^
DE: ^His^Dianthin-EGF
DMEM: Dulbecco’s modified eagle medium
ECU: Endosomal cleavable unit
EEE: Endosomal escape enhancer
EGF: Epidermal growth factor
EGFR: Epidermal growth factor receptor
IC50-value: Inhibitory concentration at 50% cell survival
IPTG: Isopropyl β-d-1-thiogalactopyranoside
LB medium: Lysogeny broth medium
MBP: Maltose-binding protein
MTT: 3-[4,5-dimethylthiazol-2-yl]-2,5 diphenyl tetrazolium bromide
Ni-NTA: Nickel nitrilotriacetic acid
OD: Optical density
PBS: Phosphate-buffered saline
RIP: Ribosome-inactivating protein
SDS-PAGE: Sodium dodecyl sulfate-polyacrylamide gel electrophoresis

## References

[1] Lee YT, Tan YJ, Oon CE (2018). Molecular targeted therapy: treating cancer with specificity. Eur J Pharmacol.

[2] Hsu JL, Hung MC (2016). The role of HER2, EGFR, and other receptor tyrosine kinases in breast cancer. Cancer Metastasis Rev.

[3] Rotow J, Bivona TG (2017). Understanding and targeting resistance mechanisms in NSCLC. Nat Rev Cancer.

[4] Roskoski R (2014). The ErbB/HER family of protein-tyrosine kinases and cancer. Pharmacol Res.

[5] El Bali M, Bakkach J, Bennani MM (2021). Colorectal Cancer: from genetic landscape to targeted therapy. J Oncol.

[6] Abourehab MAS, Alqahtani AM, Youssif BGM, Gouda AM. Globally approved EGFR inhibitors: insights into their syntheses, target kinases, biological activities, receptor interactions, and metabolism. Molecules. 2021;26(21) 10.3390/molecules26216677. 10.3390/molecules26216677 PMC8587155 34771085

[7] Sanz L, Ibanez-Perez R, Guerrero-Ochoa P, Lacadena J, Anel A. Antibody-based immunotoxins for colorectal Cancer therapy. Biomedicines. 2021;9(11) 10.3390/biomedicines9111729. 10.3390/biomedicines9111729 PMC8615520 34829955

[8] Pettinato MC. Introduction to antibody-drug conjugates. Antibodies (Basel). 2021;10(4) 10.3390/antib10040042. 10.3390/antib10040042 PMC8628511 34842621

[9] Endo Y, Tsurugi K, Lambert JM (1988). The site of action of six different ribosome-inactivating proteins from plants on eukaryotic ribosomes: the RNA N-glycosidase activity of the proteins. Biochem Biophys Res Commun.

[10] Schrot J, Weng A, Melzig MF (2015). Ribosome-inactivating and related proteins. Toxins (Basel).

[11] Gilabert-Oriol R, Weng A, Mallinckrodt B, Melzig MF, Fuchs H, Thakur M (2014). Immunotoxins constructed with ribosome-inactivating proteins and their enhancers: a lethal cocktail with tumor specific efficacy. Curr Pharm Des.

[12] von Mallinckrodt B, Thakur M, Weng A, Gilabert-Oriol R, Dürkop H, Brenner W (2014). Dianthin-EGF is an effective tumor targeted toxin in combination with saponins in a xenograft model for colon carcinoma. Future Oncol.

[13] Gilabert-Oriol R, Weng A, Trautner A, Weise C, Schmid D, Bhargava C (2015). Combinatorial approach to increase efficacy of Cetuximab, Panitumumab and Trastuzumab by dianthin conjugation and co-application of SO1861. Biochem Pharmacol.

[14] Bhargava C, Dürkop H, Zhao X, Weng A, Melzig MF, Fuchs H (2017). Targeted dianthin is a powerful toxin to treat pancreatic carcinoma when applied in combination with the glycosylated triterpene SO1861. Mol Oncol.

[15] Fuchs H. Dianthin and its potential in targeted tumor therapies. Toxins (Basel). 2019;11(10) 10.3390/toxins11100592. 10.3390/toxins11100592 PMC6832487 31614697

[16] Olsnes S, Sandvig K, Petersen OW, van Deurs B (1989). Immunotoxins--entry into cells and mechanisms of action. Immunol Today.

[17] Sparg SG, Light ME, van Staden J (2004). Biological activities and distribution of plant saponins. J Ethnopharmacol.

[18] Weng A, Thakur M, Beceren-Braun F, Bachran D, Bachran C, Riese SB (2012). The toxin component of targeted anti-tumor toxins determines their efficacy increase by saponins. Mol Oncol.

[19] Fuchs H, Niesler N, Trautner A, Sama S, Jerz G, Panjideh H, et al. Glycosylated triterpenoids as endosomal escape enhancers in targeted tumor therapies. Biomedicines. 2017;5(2) 10.3390/biomedicines5020014. 10.3390/biomedicines5020014 PMC5489800 28536357

[20] Heisler I, Keller J, Tauber R, Sutherland M, Fuchs H (2003). A cleavable adapter to reduce nonspecific cytotoxicity of recombinant immunotoxins. Int J Cancer.

[21] Heisler I, Sutherland M, Bachran C, Hebestreit P, Schnitger A, Melzig MF (2005). Combined application of saponin and chimeric toxins drastically enhances the targeted cytotoxicity on tumor cells. J Control Release.

[22] Fuchs H, Bachran C, Li T, Heisler I, Dürkop H, Sutherland M (2007). A cleavable molecular adapter reduces side effects and concomitantly enhances efficacy in tumor treatment by targeted toxins in mice. J Control Release.

[23] Oess S, Hildt E (2000). Novel cell permeable motif derived from the PreS2-domain of hepatitis-B virus surface antigens. Gene Ther.

[24] Keller J, Heisler I, Tauber R, Fuchs H (2001). Development of a novel molecular adapter for the optimization of immunotoxins. J Control Release.

[25] Smith TA, Cheyne RW (2011). Predicting tumour response to anti-HER1 therapy using medical imaging: a literature review and in vitro study of [18F]-FDG incorporation by breast cancer cells responding to cetuximab. Br J Biomed Sci.

[26] Kumar M, Joshi G, Arora S, Singh T, Biswas S, Sharma N, et al. Design and synthesis of non-covalent Imidazo [1,2-a]quinoxaline-based inhibitors of EGFR and their anti-Cancer assessment. Molecules. 2021;26(5) 10.3390/molecules26051490. 10.3390/molecules26051490 PMC7967119 33803355

[27] Schindler J, Gajavelli S, Ravandi F, Shen Y, Parekh S, Braunchweig I (2011). A phase I study of a combination of anti-CD19 and anti-CD22 immunotoxins (Combotox) in adult patients with refractory B-lineage acute lymphoblastic leukaemia. Br J Haematol.

[28] Borthakur G, Rosenblum MG, Talpaz M, Daver N, Ravandi F, Faderl S (2013). Phase 1 study of an anti-CD33 immunotoxin, humanized monoclonal antibody M195 conjugated to recombinant gelonin (HUM-195/rGEL), in patients with advanced myeloid malignancies. Haematologica..

[29] Manoukian G, Hagemeister F (2009). Denileukin diftitox: a novel immunotoxin. Expert Opin Biol Ther.

[30] Dhillon S (2018). Moxetumomab Pasudotox: First Global Approval. Drugs..

[31] Pirie CM, Liu DV, Wittrup KD (2013). Targeted cytolysins synergistically potentiate cytoplasmic delivery of gelonin immunotoxin. Mol Cancer Ther.

[32] Polli JR, Chen P, Bordeau BM, Balthasar JP (2022). Targeted delivery of endosomal escape peptides to enhance immunotoxin potency and anti-cancer efficacy. AAPS J.

[33] Thomas G (2002). Furin at the cutting edge: from protein traffic to embryogenesis and disease. Nat Rev Mol Cell Biol.

[34] Bachran D, Schneider S, Bachran C, Urban R, Weng A, Melzig MF (2010). Epidermal growth factor receptor expression affects the efficacy of the combined application of saponin and a targeted toxin on human cervical carcinoma cells. Int J Cancer.

[35] Fischer A, Wolf I, Fuchs H, Masilamani AP, Wolf P. Pseudomonas exotoxin a based toxins targeting epidermal growth factor receptor for the treatment of prostate Cancer. Toxins (Basel). 2020;12(12) 10.3390/toxins12120753. 10.3390/toxins12120753 PMC7761469 33260619

[36] Berstad MB, Cheung LH, Berg K, Peng Q, Fremstedal AS, Patzke S (2015). Design of an EGFR-targeting toxin for photochemical delivery: in vitro and in vivo selectivity and efficacy. Oncogene..

[37] Bachran C, Weng A, Bachran D, Riese SB, Schellmann N, Melzig MF (2010). The distribution of saponins in vivo affects their synergy with chimeric toxins against tumours expressing human epidermal growth factor receptors in mice. Br J Pharmacol.

[38] Heisler I, Keller J, Tauber R, Sutherland M, Fuchs H (2002). A colorimetric assay for the quantitation of free adenine applied to determine the enzymatic activity of ribosome-inactivating proteins. Anal Biochem.

[39] Balin-Gauthier D, Delord JP, Rochaix P, Mallard V, Thomas F, Hennebelle I (2006). In vivo and in vitro antitumor activity of oxaliplatin in combination with cetuximab in human colorectal tumor cell lines expressing different level of EGFR. Cancer Chemother Pharmacol.

[40] Hall RE, Birrell SN, Tilley WD, Sutherland RL (1994). MDA-MB-453, an androgen-responsive human breast carcinoma cell line with high level androgen receptor expression. Eur J Cancer.

[41] Niesler N, Arndt J, Silberreis K, Fuchs H (2020). Generation of a soluble and stable apoptin-EGF fusion protein, a targeted viral protein applicable for tumor therapy. Protein Expr Purif.

[42] Gilabert-Oriol R, Thakur M, Haussmann K, Niesler N, Bhargava C, Görick C (2016). Saponins from Saponaria officinalis L. augment the efficacy of a rituximab-immunotoxin. Planta Med.

[43] Mosmann T (1983). Rapid colorimetric assay for cellular growth and survival: application to proliferation and cytotoxicity assays. J Immunol Methods.

