# Supplementary material for: A cleavable peptide adapter augments the activity of targeted toxins in combination with the glycosidic endosomal escape enhancer SO1861
Source: BMC Biotechnol. 2024 Apr 29;24:24. doi: 10.1186/s12896-024-00854-5 (PMC11057116; doi:10.1186/s12896-024-00854-5)
Supplement: Supplementary file 1 — Supplementary Material 1. [file 12896_2024_854_MOESM1_ESM.pdf]

<u>**Overview of additional files**</u>

**Additional file 1**	Uncropped gels and blots to figure 2
**Additional file 2**	Coomassie of DAES purification by Strep-Tactin affinity chromatography
**Additional file 3**	Uncropped gels to Figure 3
**Additional file 4**	Data of DAES incubation alongside caspase-3 and furin
**Additional file 5**	Data of adenine release assays
**Additional file 6**	Data of cytotoxicity assays
**Additional file 7**	Detailed results of cytotoxicity assays
**Additional file 8**	Graphic comparison of the cytotoxicity towards MDA-MB-453 cells with the cytotoxicity towards HCT 116 cells
**Additional file 9**	Uncropped blots to figure 6
**Additional file 10**	Data of incubation of DE and DAES in human plasma
**Additional file 11**	EGFR expression in HCT 116 and MDA-MB-453 cells
**Additional file 12**	Cytotoxicity assays with NIH3T3 and HER14 cells

Additional file 1: Uncropped gels and blots to Figure 2

This additional file includes the uncropped SDS-PAGE gels and blots that were used for figure 2.

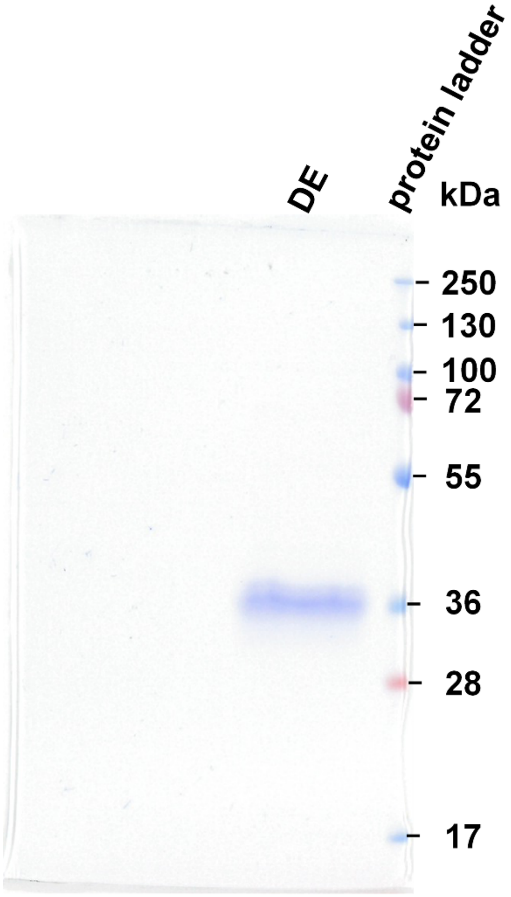

Additional fig. 1 Purified DE, Coomassie

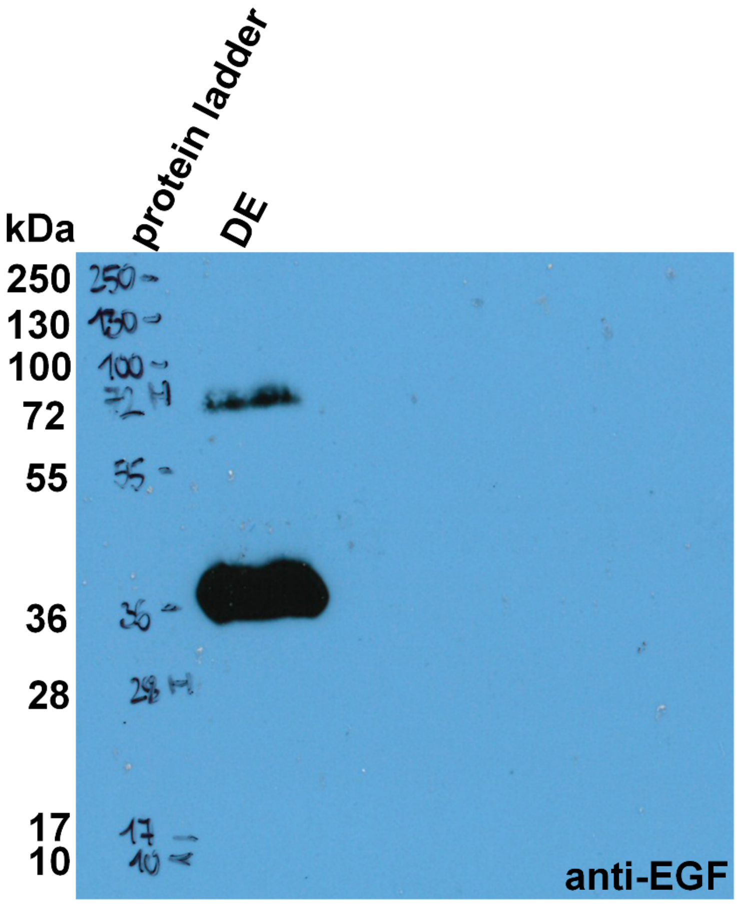

Additional fig. 2 Purified DE, Western blot with anti-EGF

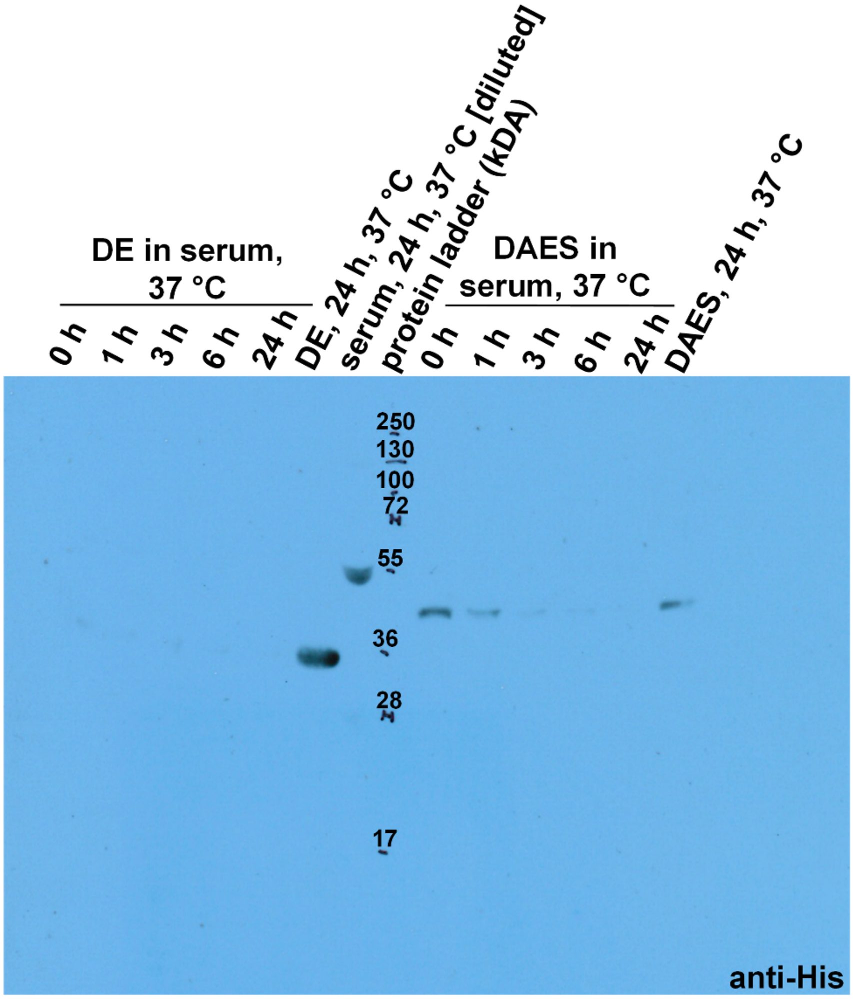

Additional fig. 3 Purified DE (lane 6), Western blot with anti-His

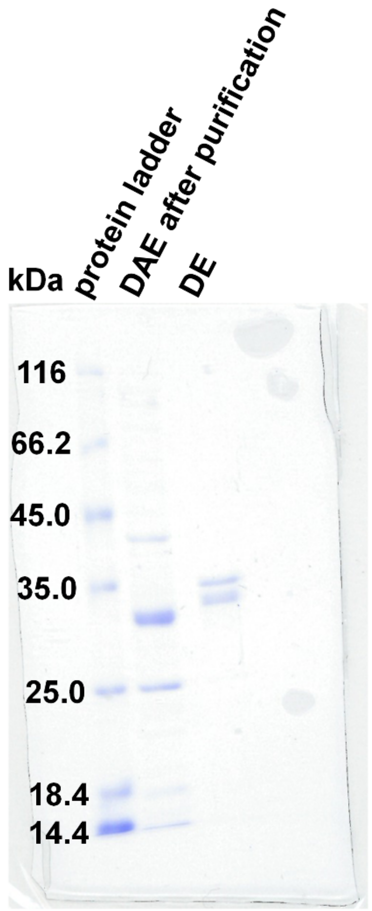

Additional fig. 4 Purified DAE, Coomassie

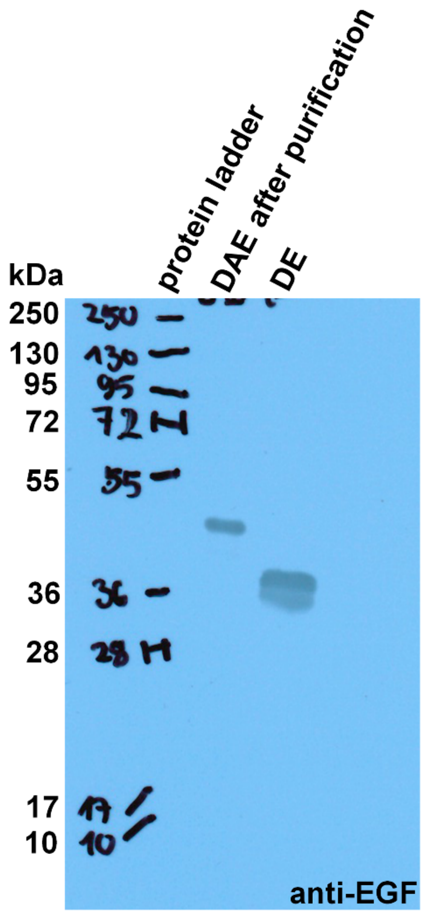

Additional fig. 5 Purified DAE, Western blot with anti-EGF

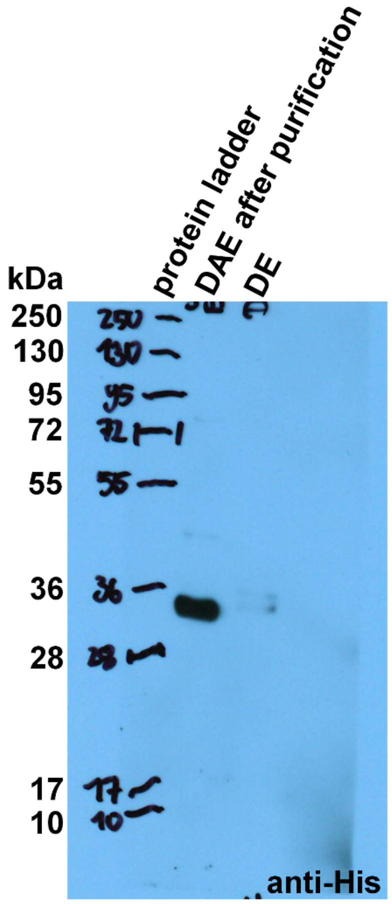

Additional fig. 6 Purified DAE, Western blot with anti-His

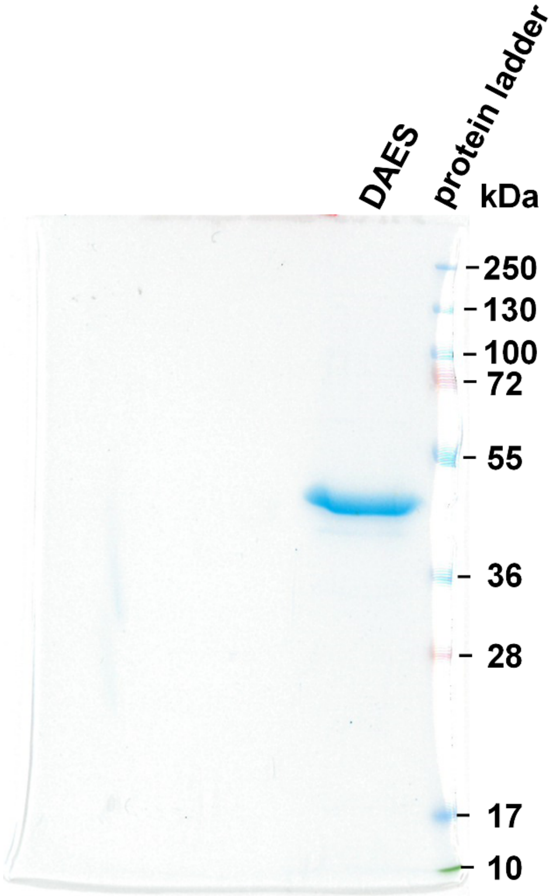

Additional fig. 7 Purified DAES, Coomassie

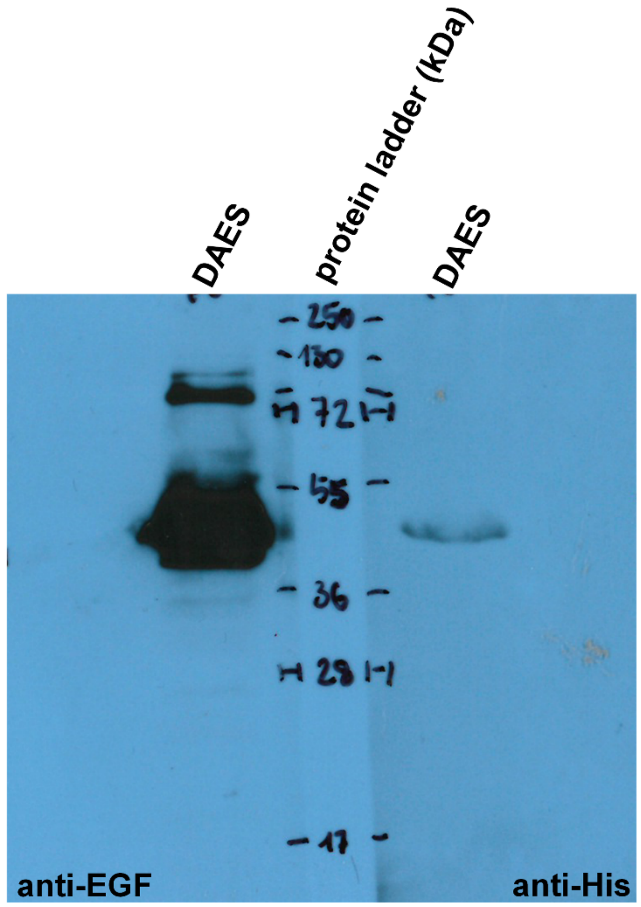

Additional fig. 8 Purified DAES, Western blots with anti-EGF and anti-His

Additional file 2 Coomassie of DAES purification by Strep-Tactin affinity chromatography

The process of DAES purification by Strep-Tactin affinity chromatography is shown in Coomassie.

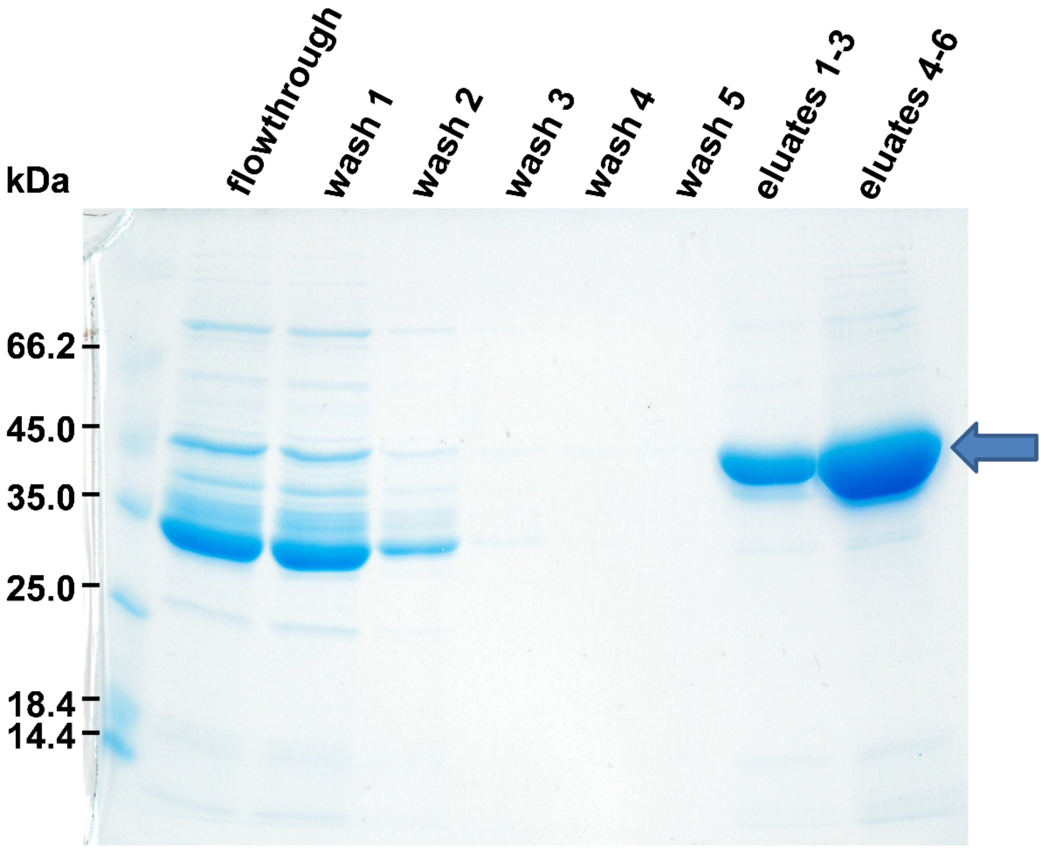

Additional fig. 9 The process of DAES purification by Strep-Tactin-affinity chromatography is shown in Coomassie. After initial purification with NiNTA, cleaved DAES (at about 34 kDa) and other protein residues were visible in the flowthrough of the Strep-Tactin-column (lane "flowthrough") and wash fractions (lanes "wash 1" to "wash 5" corresponding to five wash steps with PBS) of the Strep-Tactin column. In contrast, intact protein (blue arrow) bound to the column and was eluted with desthiobiotin in six elution steps. Elution fractions 13 and 46 were pooled.

Additional file 3 Uncropped gels to Figure 3

This additional file includes the uncropped SDS-PAGE gels that were used for figure 3.

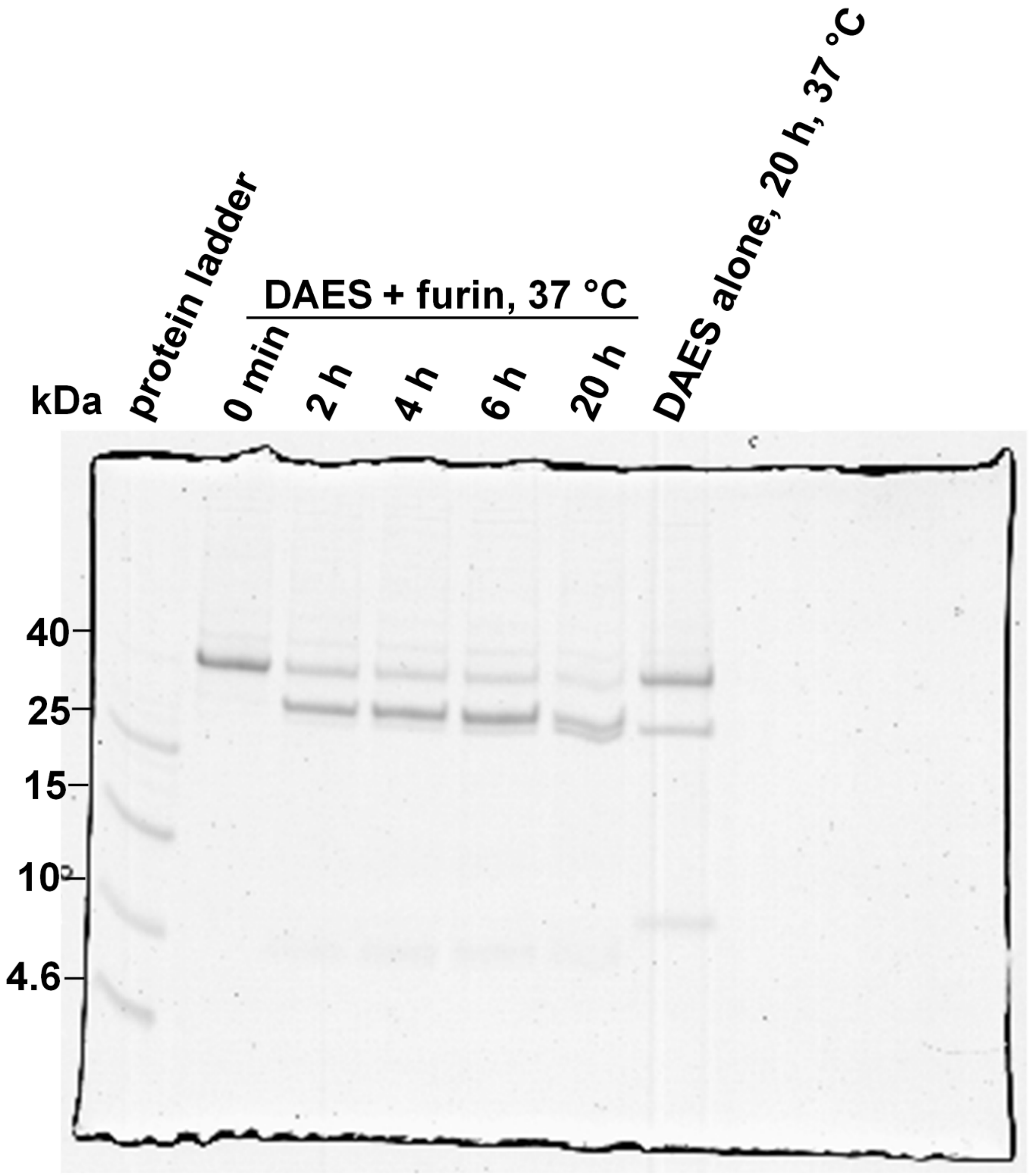

Additional fig. 10 Incubation of DAES alongside furin

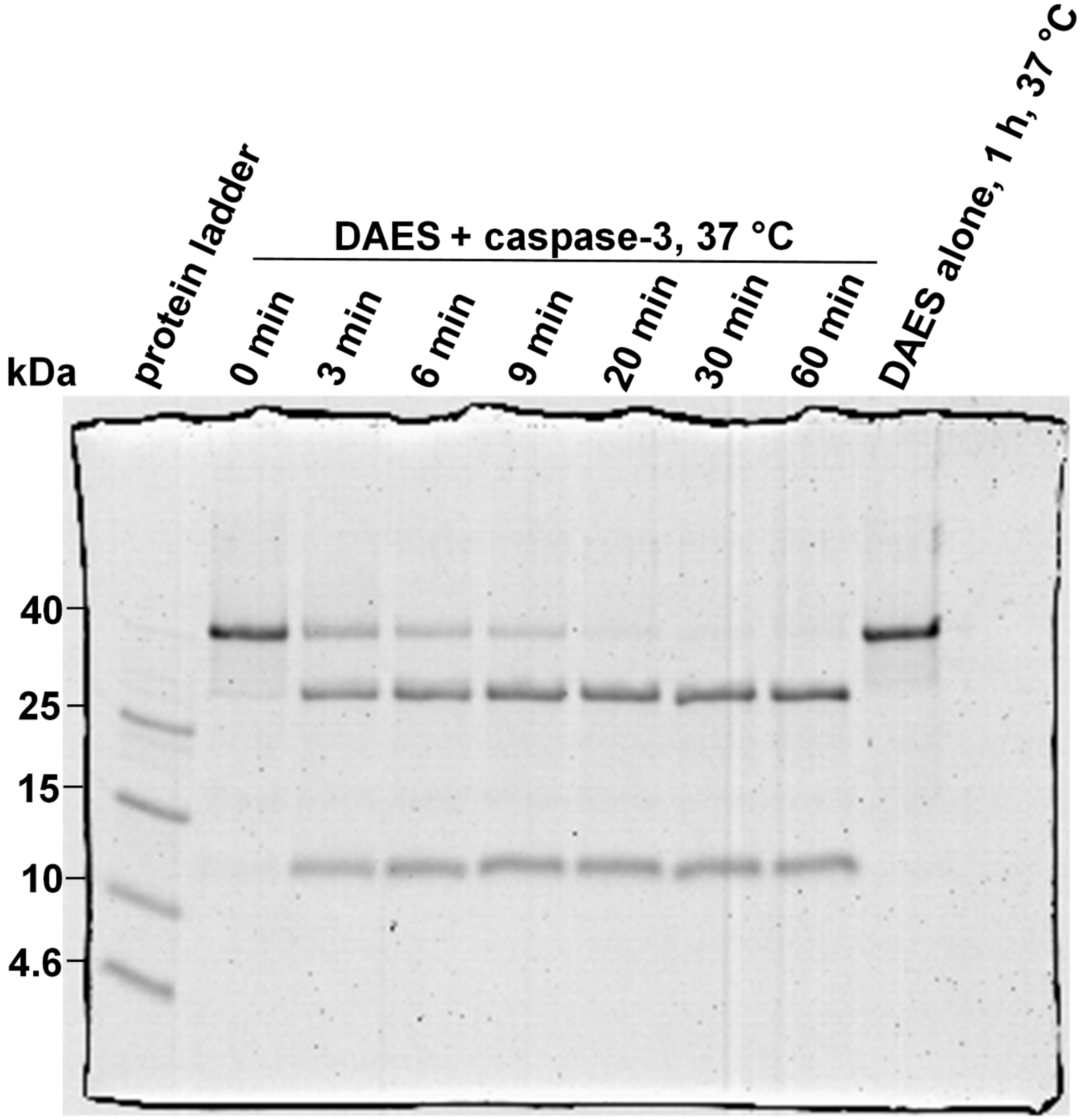

Additional fig. 11 Incubation of DAES alongside caspase-3

Additional file 4 Data of DAES incubation alongside caspase-3 and furin

This file shows the original data resulting from the quantification of band intensities after incubation of DAES alongside caspase-3 or furin.

Additional Table 1 Incubation alongside caspase-3

		**percentage of total band intensity**		
		**DAES**	**large fragment**	**small fragment**
0 min	experiment 1	0.968193	0.031806616	
	experiment 2	0.912596	0.038560411	0.048843188
	experiment 3	0.947955	0.024783147	0.027261462
3 min	experiment 1	0.277228	0.420792079	0.301980198
	experiment 2	0.331375	0.347826087	0.32079906
	experiment 3	0.35169	0.331664581	0.316645807
6 min	experiment 1	0.174432	0.463560335	0.362007168
	experiment 2	0.199301	0.459207459	0.341491841
	experiment 3	0.18712	0.461725395	0.351154313
9 min	experiment 1	0.11385	0.517605634	0.368544601
	experiment 2	0.120231	0.519075145	0.360693642
	experiment 3	0.126761	0.503521127	0.36971831
20 min	experiment 1	0.042453	0.555424528	0.402122642
	experiment 2	0.031323	0.569605568	0.399071926
	experiment 3	0.026448	0.573047859	0.400503778
30 min	experiment 1	0.027348	0.577883472	0.394768133
	experiment 2	0.021004	0.568261377	0.410735123
	experiment 3	0.021152	0.564042303	0.41480611
1 h	experiment 1	0.017626	0.623971798	0.35840188
	experiment 2	0.017202	0.597477064	0.385321101
	experiment 3	0.021505	0.603345281	0.375149343

Additional Table 2 Incubation alongside furin

		percentage of total band intensity		
		**DAES**	**large fragment**	**small fragment**
0 min	experiment 1	0.980653	0.019347037	
	experiment 2	0.995068	0.004932182	
	experiment 3	0.993498	0.006501951	
2 h	experiment 1	0.3	0.613333333	0.086666667
	experiment 2	0.311284	0.666666667	0.022049287
	experiment 3	0.338812	0.623261694	0.037926675
4 h	experiment 1	0.170878	0.736462094	0.092659446
	experiment 2	0.20073	0.721411192	0.077858881
	experiment 3	0.209738	0.725343321	0.064918851
6 h	experiment 1	0.151765	0.765882353	0.082352941
	experiment 2	0.157248	0.773955774	0.068796069
	experiment 3	0.169951	0.772167488	0.057881773
20 h	experiment 1	0.107417	0.803069054	0.089514066
	experiment 2	0.127321	0.778514589	0.094164456
	experiment 3	0.136662	0.814717477	0.048620237
DAES without furin, 20 h	experiment 1	0.62933	0.249422633	0.121247113
	experiment 2	0.609871	0.247943596	0.142185664
	experiment 3	0.597826	0.266908213	0.1352657

Additional file 5 Data of adenine release assays

This file shows the original data resulting from adenine release assays.

Additional Table 3 Generation of a standard curve via measurement of samples with defined adenine concentration

**adenine concentration**		**absorption at defined wavelength**		
		**A260**	**A300 (reference)**	**A260-A300**
320 pmol/µL	measurement 1	3.525	0.019	3.506
	measurement 2	3.491	0.026	3.465
	measurement 3	3.506	0.01	3.496
160 pmol/µL	measurement 1	1.739	0.027	1.712
	measurement 2	1.704	0.001	1.703
	measurement 3	1.742	0.023	1.719
80 pmol/µL	measurement 1	0.93	0.054	0.876
	measurement 2	0.895	0.031	0.864
	measurement 3	0.866	-0.004	0.87
40 pmol/µL	measurement 1	0.526	0.042	0.484
	measurement 2	0.473	0.07	0.403
	measurement 3	0.478	0.04	0.438
20 pmul/µL	measurement 1	0.272	0.043	0.229
	measurement 2	0.244	0.036	0.208
	measurement 3	0.226	0.027	0.199
10 pmol/µL	measurement 1	0.127	0.026	0.101
	measurement 2	0.125	0.026	0.099
	measurement 3	0.108	0.009	0.099

Additional Table 4 Measurement of the absorption of the different samples

sample		absorption at defined wavelength		
		**A260**	**A300 (reference)**	**A260-A300**
DAES, first experiment	measurement 1	0.295	0.024	0.271
	measurement 2	0.385	0.026	0.359
	measurement 3	0.399	0.05	0.349
DAES, second experiment	measurement 1	0.358	0.037	0.321
	measurement 2	0.361	0.041	0.32
	measurement 3	0.348	0.017	0.331
DAES, third experiment	measurement 1	0.362	0.019	0.343
	measurement 2	0.381	0.06	0.321
	measurement 3	0.385	0.066	0.319
DAES after caspase-3, first experiment	measurement 1	1.329	0.114	1.215
	measurement 2	1.328	0.082	1.246
	measurement 3	1.372	0.141	1.231
DAES after caspase-3, second experiment	measurement 1	1.638	0.135	1.503
	measurement 2	1.589	0.096	1.493
	measurement 3	1.611	0.13	1.481
DAES after caspase-3, third experiment	measurement 1	1.345	0.11	1.235
	measurement 2	1.327	0.1	1.227
	measurement 3	1.339	0.113	1.226
DAES after furin, first experiment	measurement 1	0.571	0.025	0.546
	measurement 2	0.581	0.053	0.528
	measurement 3	0.569	0.025	0.544
DAES after furin, second experimen	measurement 1	0.498	0.049	0.449
	measurement 2	0.493	0.027	0.466
	measurement 3	0.483	0.023	0.46
DAES after furin, third experimen	measurement 1	0.503	0.045	0.458
	measurement 2	0.496	0.04	0.456
	measurement 3	0.528	0.089	0.439
DAES after furin, fourth experimen	measurement 1	0.559	0.056	0.503
	measurement 2	0.514	0.036	0.478
	measurement 3	0.524	0.023	0.501
DAES after furin, fifth experimen	measurement 1	0.693	0.028	0.665
	measurement 2	0.71	0.054	0.656
	measurement 3	0.692	0.057	0.635
BSA, first experiment	measurement 1	0.157	0.037	0.12
	measurement 2	0.169	0.056	0.113
	measurement 3	0.16	0.041	0.119
BSA, second experiment	measurement 1	0.323	0.073	0.25
	measurement 2	0.269	0.044	0.225
	measurement 3	0.324	0.084	0.24

BSA, third experiment	measurement 1	0.192	0.074	0.118
	measurement 2	0.182	0.067	0.115
	measurement 3	0.153	0.012	0.141
blank, first experiment	measurement 1	0.138	0.028	0.11
	measurement 2	0.151	0.044	0.107
	measurement 3	0.153	0.044	0.109
blank, second experiment	measurement 1	0.146	0.032	0.114
	measurement 2	0.155	0.071	0.084
	measurement 3	0.161	0.058	0.103
blank, third experiment	measurement 1	0.179	0.057	0.122
	measurement 2	0.165	0.037	0.128
	measurement 3	0.172	0.05	0.122

Additional file 6 Data of cytotoxicity assays

This file shows the original data resulting from cytotoxicity assays in graphic format.

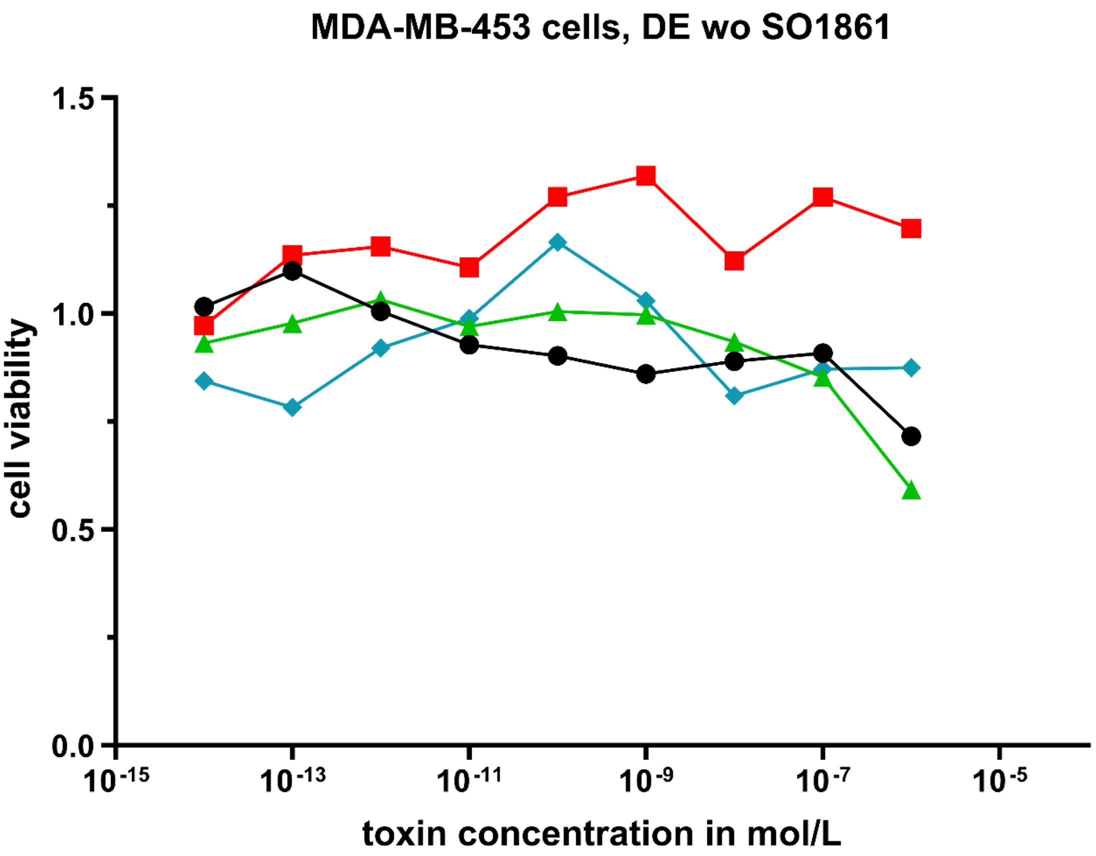

Additional fig. 12 Data of cytotoxicity assays, MDA-MB-453 cells, DE without SO1861

Each curve represents one replicate.

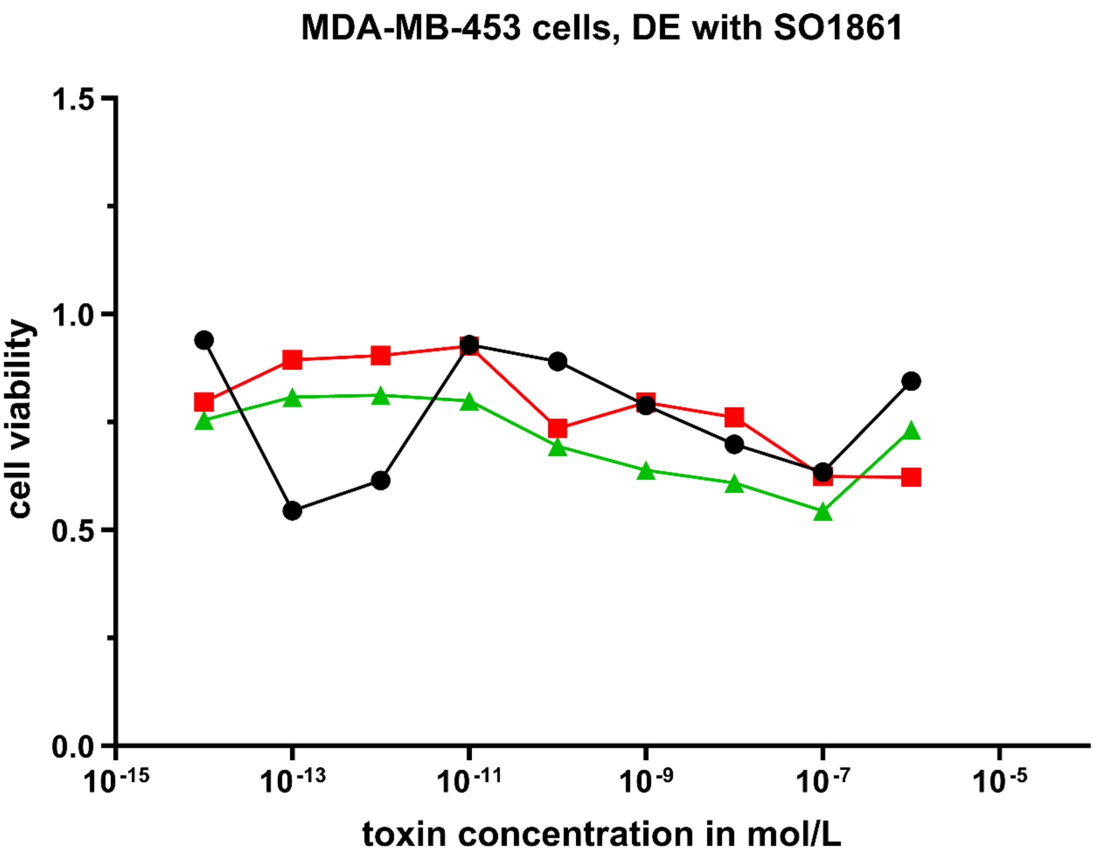

Additional fig. 13 Data of cytotoxicity assays, MDA-MB-453 cells, DE with SO1861

Each curve represents one replicate.

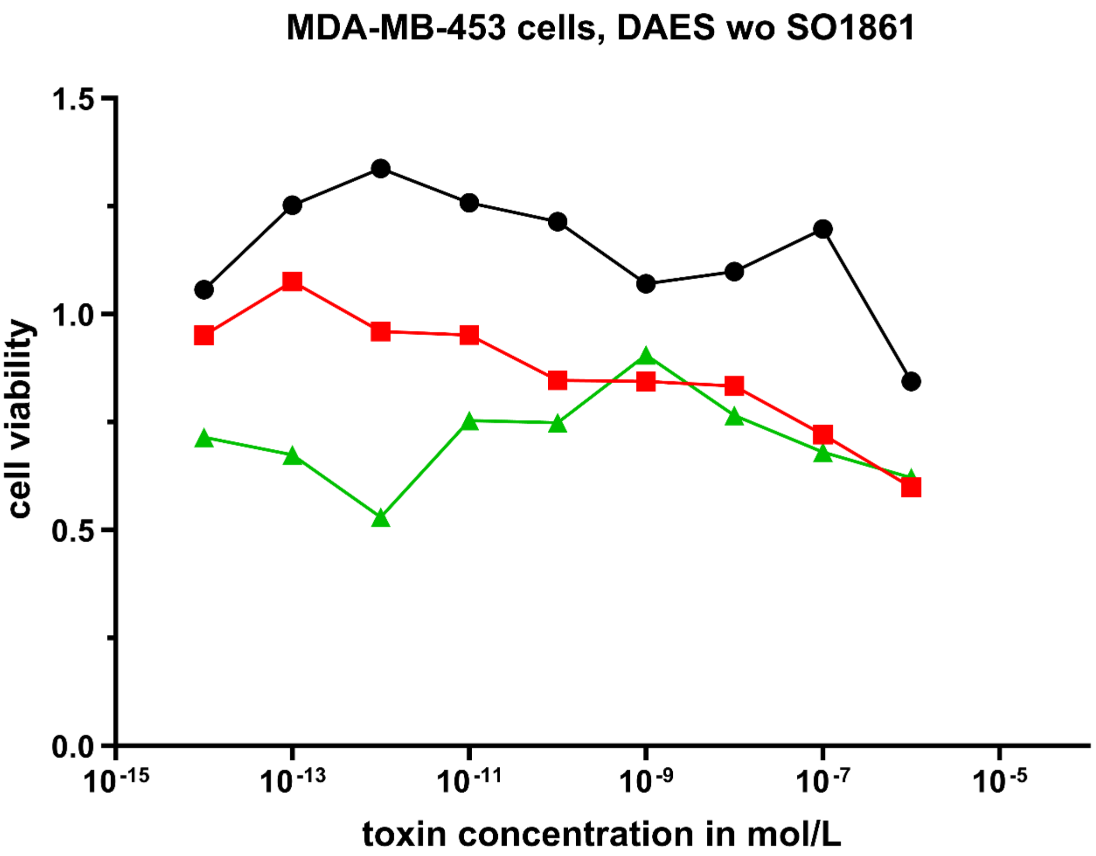

Additional fig. 14 Data of cytotoxicity assays, MDA-MB-453 cells, DAES wo SO1861

Each curve represents one replicate.

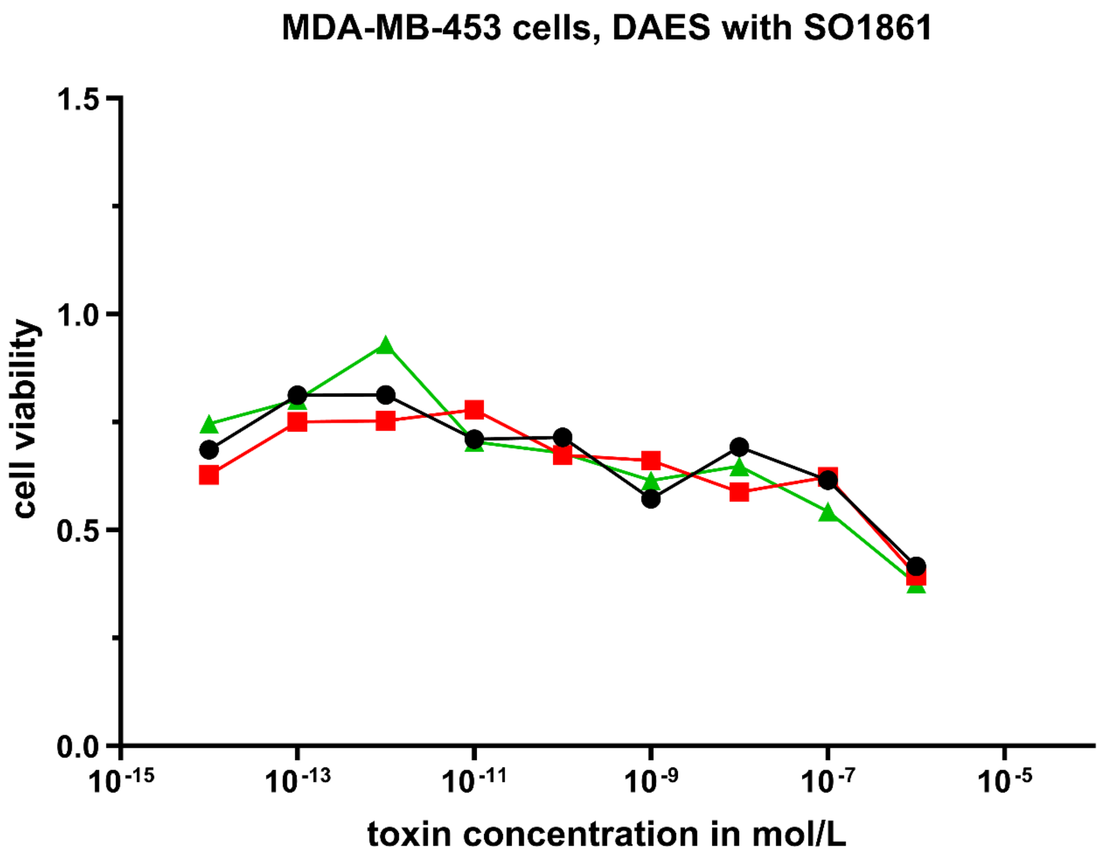

Additional fig. 15 Data of cytotoxicity assays, MDA-MB-453 cells, DAES with SO1861

Each curve represents one replicate.

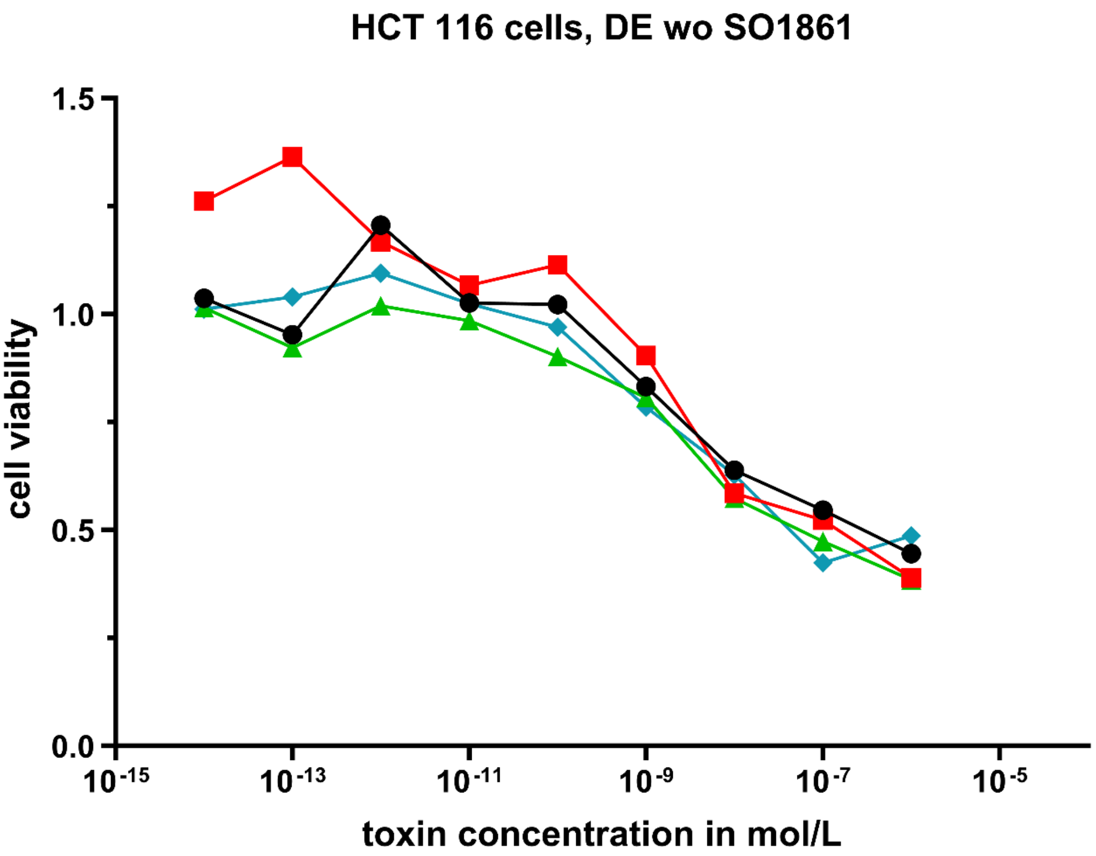

Additional fig. 16 Data of cytotoxicity assays, HCT 116 cells, DE wo SO1861

Each curve represents one replicate.

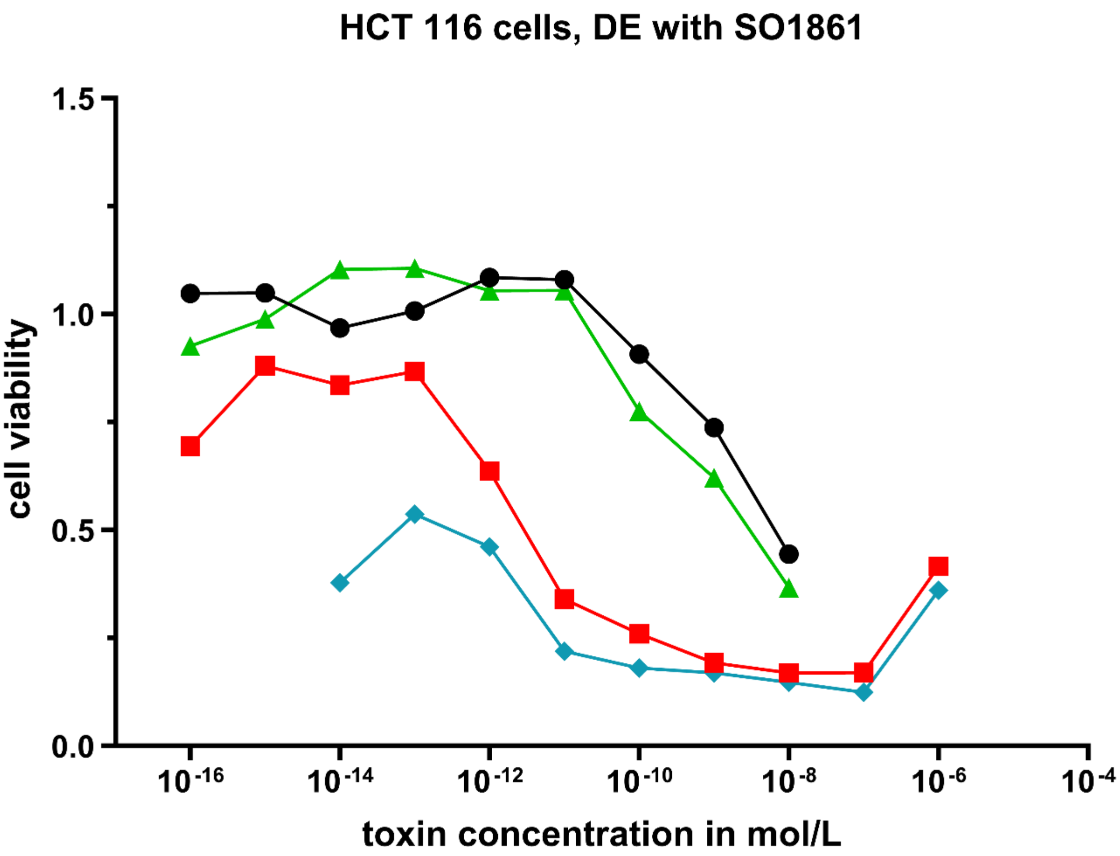

Additional fig. 17 Data of cytotoxicity assays, HCT 116 cells, DE with SO1861

Each curve represents one replicate.

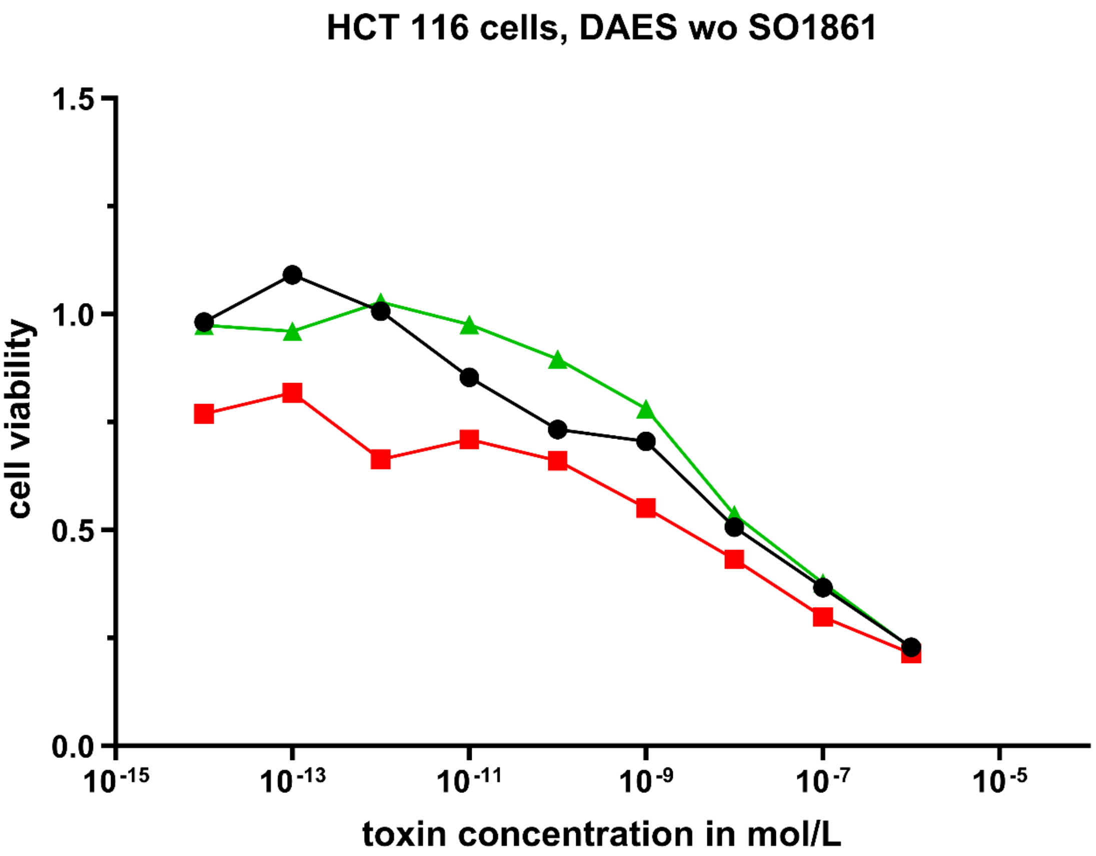

Additional fig. 18 Data of cytotoxicity assays, HCT 116 cells, DAES wo SO1861

Each curve represents one replicate.

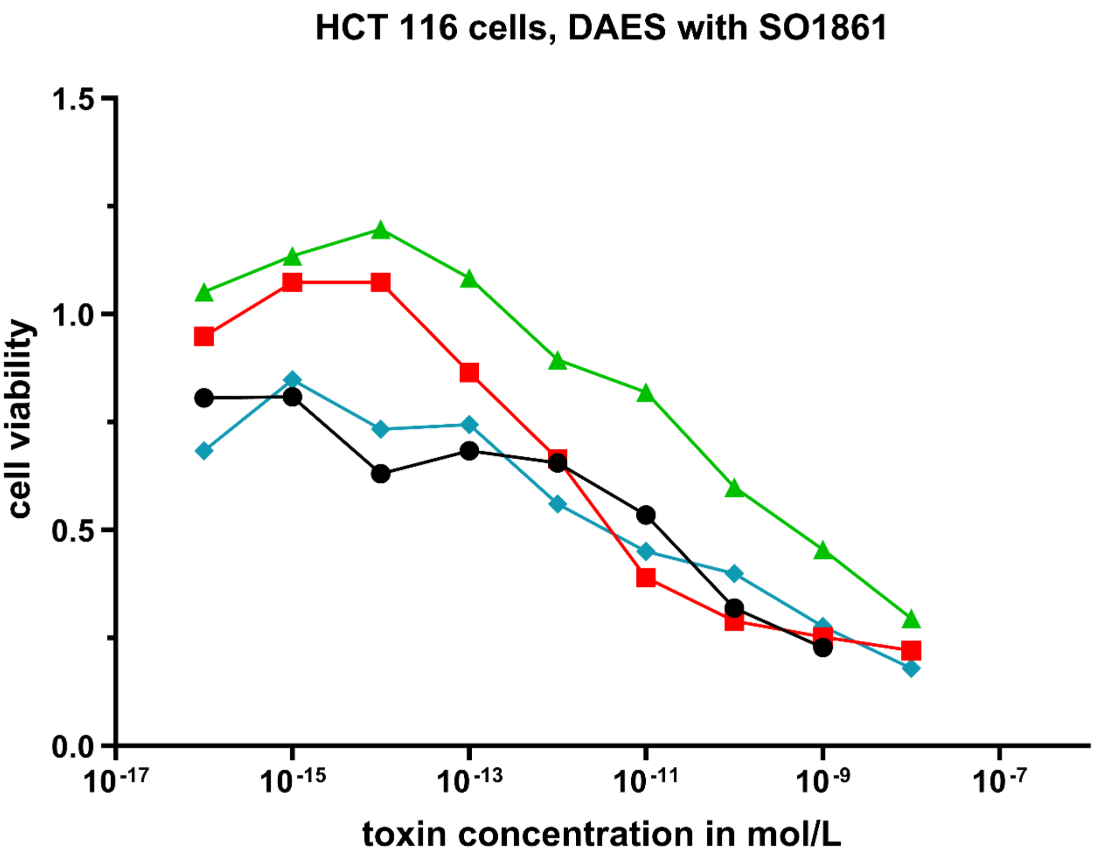

Additional fig. 19 Data of cytotoxicity assays, HCT 116 cells, DAES with SO1861

Each curve represents one replicate.

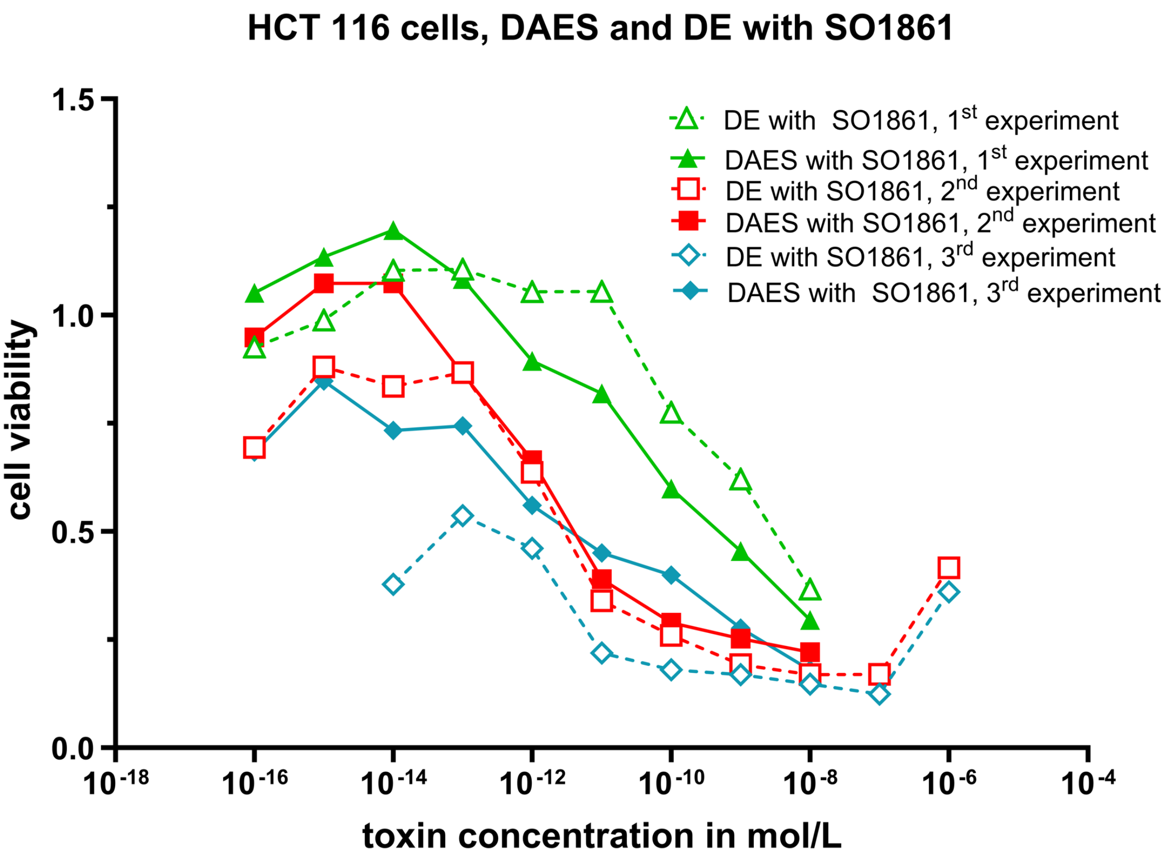

Additional fig. 20 Data of cytotoxicity assays, HCT 116 cells, DE and DAES with SO1861

The curves are copied from Additional figures 17 and 19, but now coloured according to the individual experiments to show the effect of the ancillary conditions on the result. Each curve represents one replicate.

Additional file 7 Results of cytotoxicity assays

IC_{50}-values, enhancement factors and gain in receptor specificity

This file contains tables reporting the IC_{50}-values, the enhancement factors and the gain in receptor specificity of the cytotoxicity assays with HCT 116 and MDA-MB-453 cells in detail.

Additional Table 5. IC_{50}-values [nM] and 95% confidence interval for DE and DAES ± SO1861, including corresponding enhancement factors, HCT 116 cells

HCT 116 cells	– SO1861		+ SO1861		enhancement through SO1861[a]
**DE**	130	*+ 220*<br>*– 70*	0.3	*+ 5.7*<br>*– 0.3*	**430**
**DAES**	11	*+ 19*<br>*– 7*	0.03	*+ 0.09*<br>*– 0.02*	**370**
**enhancement through A2**[a]	**12**		**10**		**4,300**[b]

[a] Each enhancement factor is calculated by division of the corresponding IC_{50}-values.

[b] enhancement factor of combination between A2 and SO1861 in comparison to DE alone

Additional Table 6. IC_{50}-values [nM] and 95% confidence interval for DE and DAES ± SO1861, including corresponding enhancement factors, MDA-MB-453 cells

MDA-MB-453 cells	– SO1861	+ SO1861	enhancement through SO1861[a]
**DE**	20,000	not determinable	**not determinable**
**DAES**	5,800	230	**25**
**enhancement through A2[a]**	**3.4**	**not determinable**	**87[b]**

[a] Each enhancement factor is calculated by division of the corresponding IC_{50}-values.

[b] enhancement factor of combination between A2 and SO1861 in comparison to DE alone

[c] Due to missing cytotoxicity for off-target cells, the 95% confidence intervals could not be calculated for MDA-MB-453 cells. The corresponding IC_{50}-values are approximate values calculated by four-parameter-regression. The IC_{50}-value for DE + SO1861 was not determinable.

Additional Table 7. Receptor target indices for DE and DAES ± SO1861, including corresponding gain in receptor specificity

	- SO1861	+ SO1861	gain in receptor specificity through SO1861
**DE**	150	not determinable	**not determinable**
**DAES**	530	7,700	**15**
**gain in receptor specificity through A2**	**3.5**	**not determinable**	**51[c]**

[a] Receptor target index = IC_{50} (MDA-MB-453 cells) / corresponding IC_{50} (HCT 116 cells); Basically, this table is obtained by dividing the values of the lower part of Table IIA by the corresponding values of the upper part.

[b] Due to missing cytotoxicity for off-target MDA-MB-453 cells, the receptor target indices are approximate values.

[c] Gain in receptor specificity is calculated by division of two receptor target indices.

[d] gain in receptor specificity concerning the combination between the A2 and SO1861 in comparison to DE alone

Additional file 8 Comparison of MDA-MB-453 and HCT 116 cells

This file shows graphs that allow ideal comparison of the cytotoxicity towards MDA-MB-453 cells with the cytotoxicity towards HCT 116 cells.

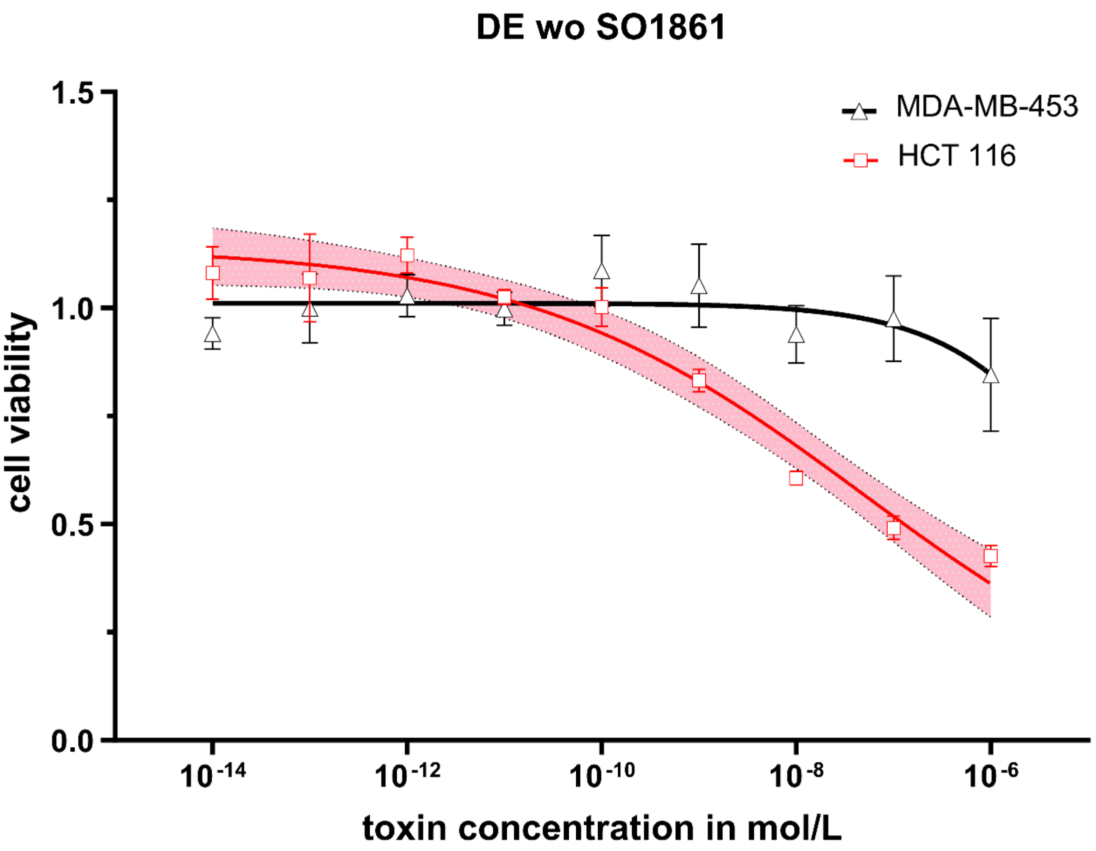

Additional fig. 21 Comparison of MDA-MB-453 and HCT 116 cells, DE wo SO1861

Further details can be found in the Figure legend of Figure 5.

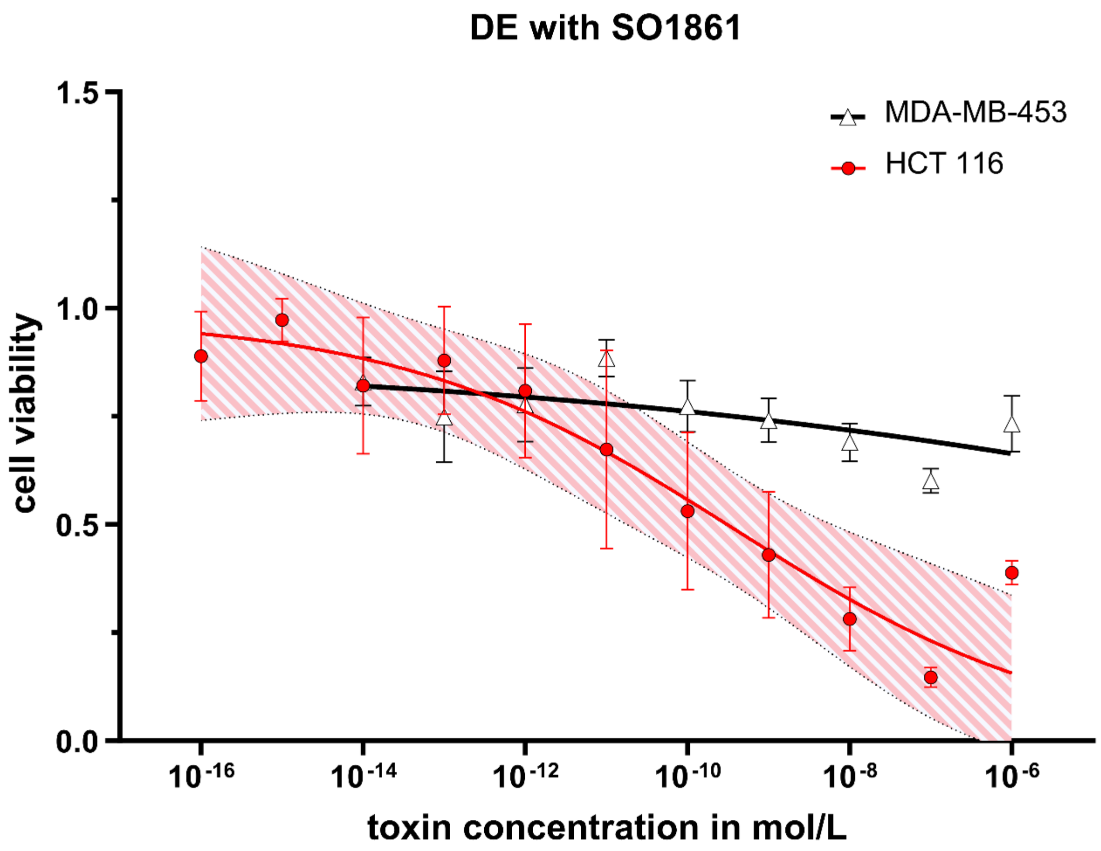

Additional fig. 22 Comparison of MDA-MB-453 and HCT 116 cells, DE with SO1861

Further details can be found in the Figure legend of Figure 5.

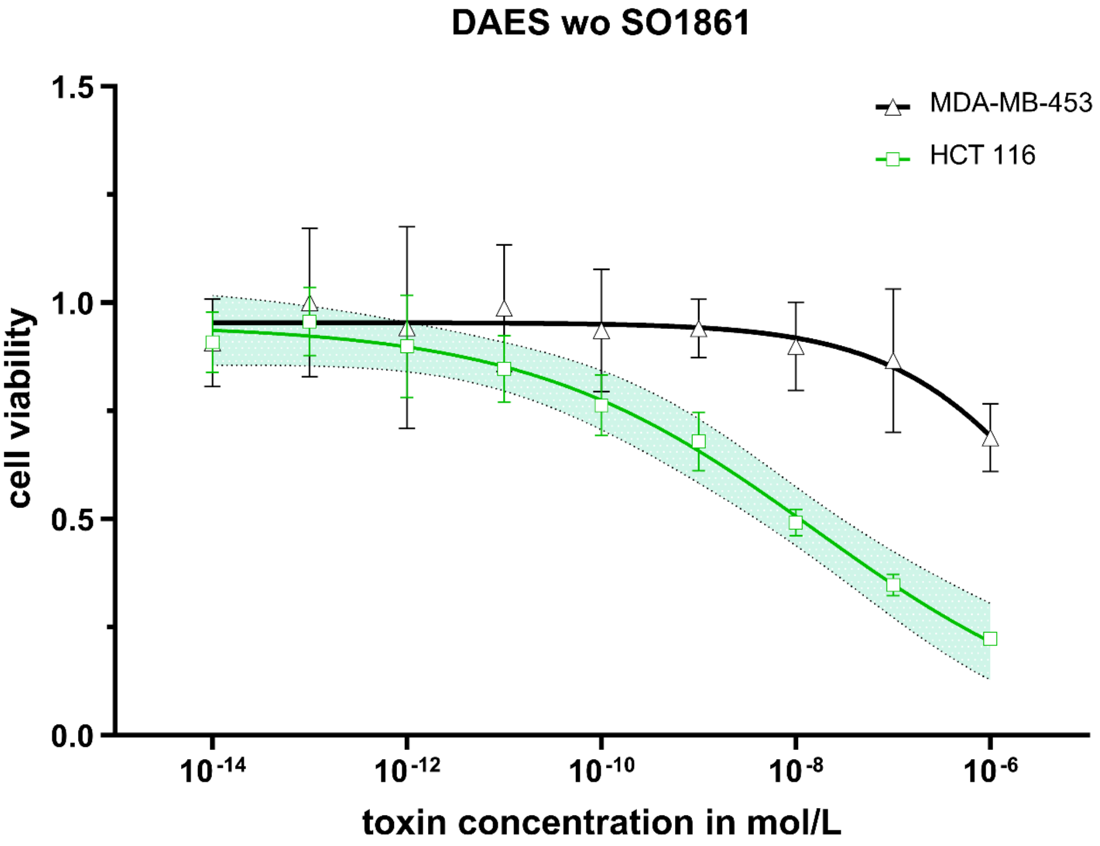

Additional fig. 23 Comparison of MDA-MB-453 and HCT 116 cells, DAES wo SO1861

Further details can be found in the Figure legend of Figure 5.

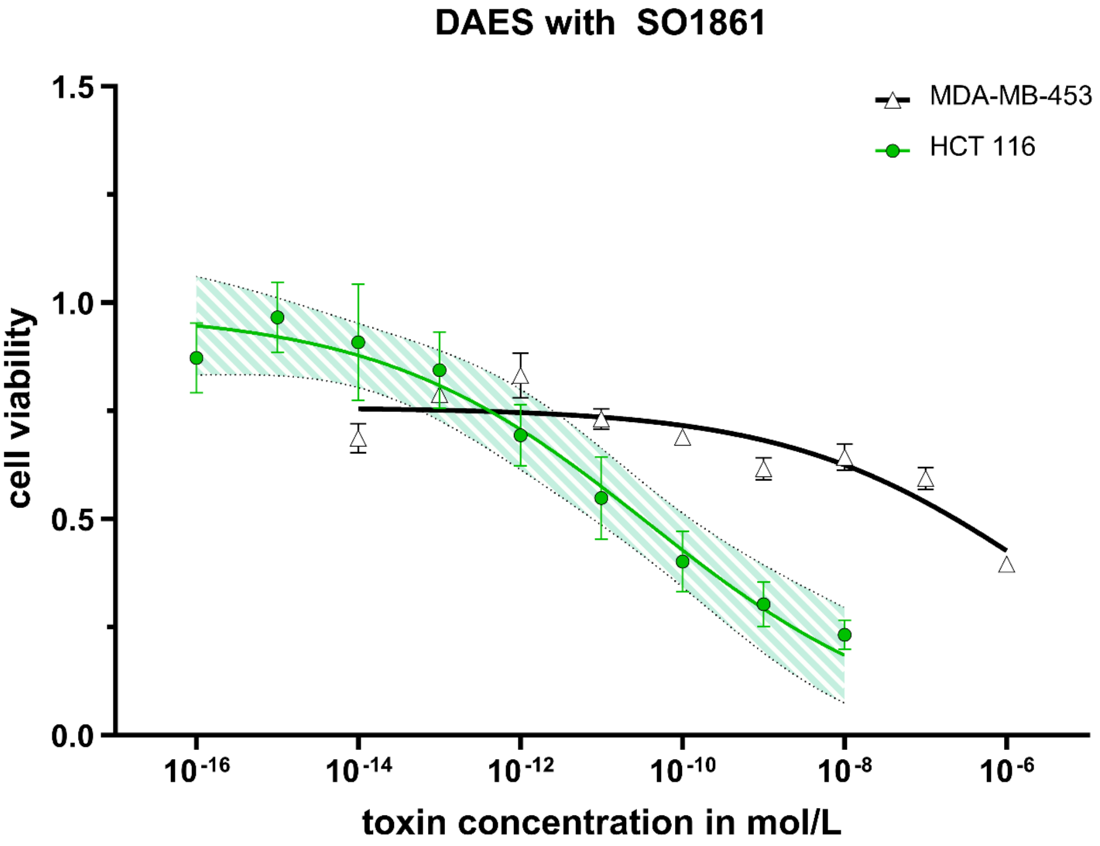

Additional fig. 24 Comparison of MDA-MB-453 and HCT 116 cells, DAES with SO1861

Further details can be found in the Figure legend of Figure 5.

Additional file 9 Uncropped blots to figure 6

This additional file includes the uncropped Western blots that were used for figure 6.

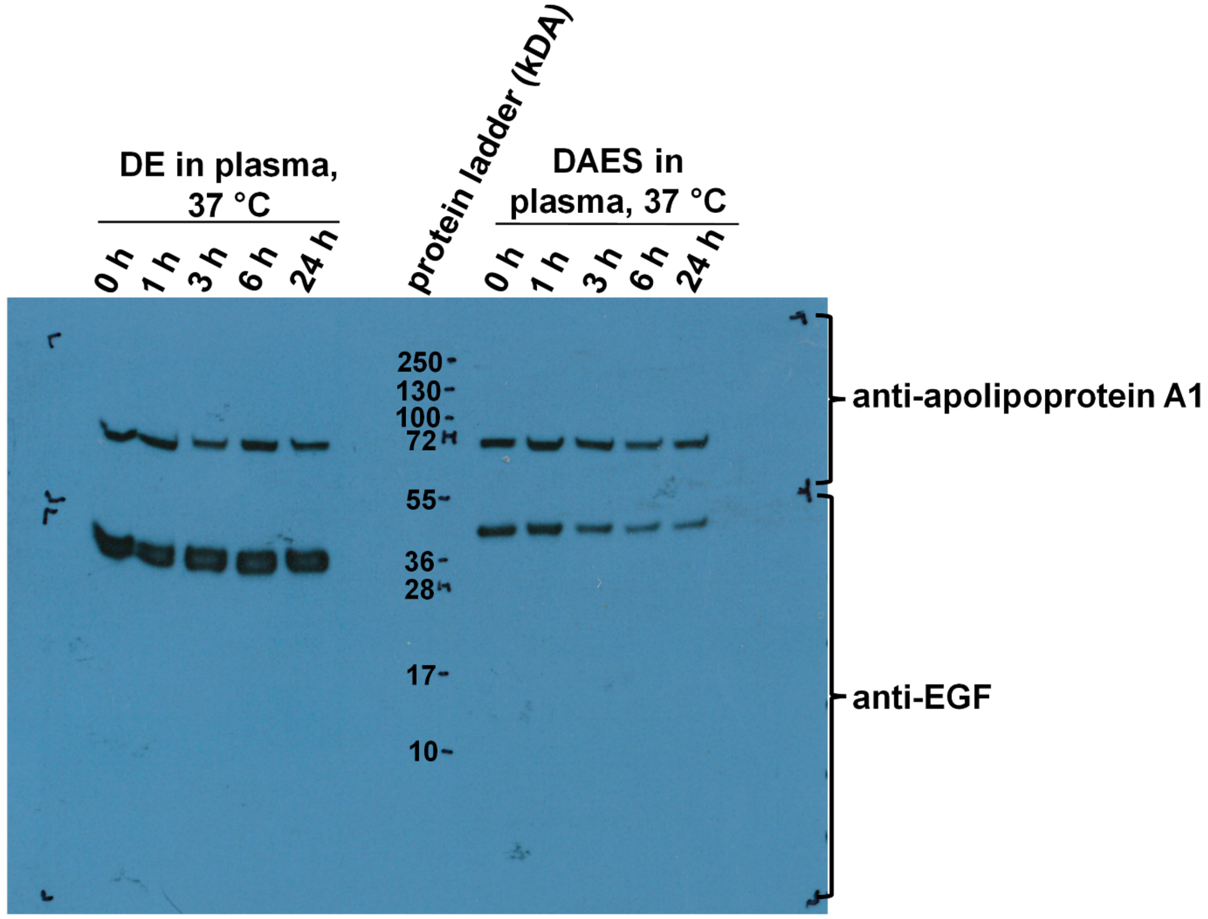

Additional fig. 25 Incubation of DE in plasma (lane 1 to 5)

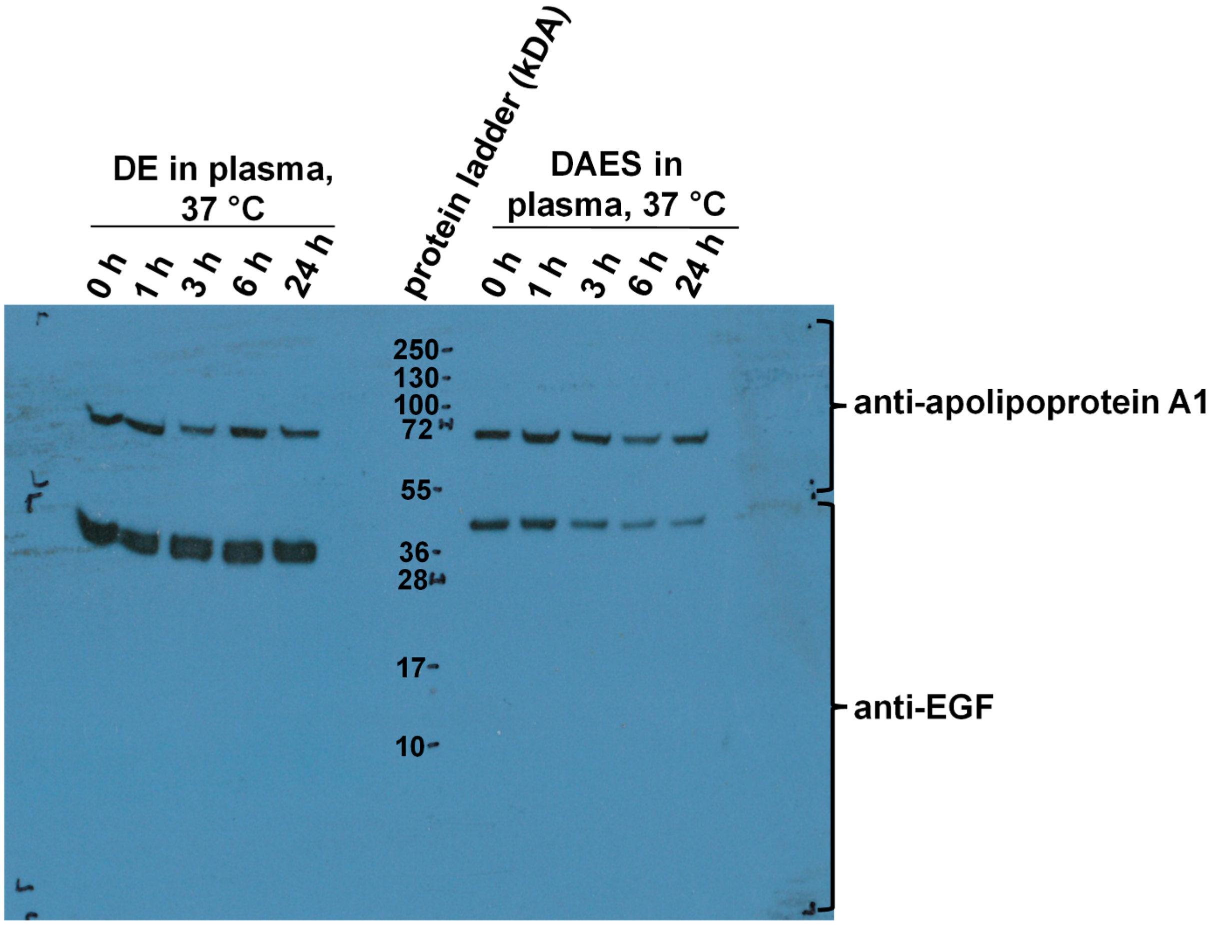

Additional fig. 26 Incubation of DAES in plasma (lane 8 to 12)

Additional file 10 Data of incubation of DE and DAES in human plasma

This file shows the original data resulting from the quantification of band intensities after incubation of DE and DAES in human plasma.

Additional Table 8 Incubation of DE in human plasma

	**quantification of band intensities via area under the curve**					
	**experiment 1**		**experiment 2**		**experiment 3**	
	DE	MBP apolipoprotein A1	DE	MBP apolipoprotein A1	DE	MBP apolipoprotein A1
0 h	27,676,756	11,164,288	24,239,886	12,925,409	25,315,756	4,159,125
1 h	19,392,016	10,677,045	17,241,530	15,577,945	22,578,501	3,447,104
3 h	26,913,865	6,135,125	20,898,844	12,229,024	30,487,844	1,226,941
6 h	24,272,551	11,009,782	18,233,279	8,764,924	16,595,480	1,762,083
24 h	24,783,329	7,262,953	22,278,614	14,393,459	25,783,279	2,913,104

Additional Table 9 Incubation of DAES in human plasma

	**quantification of band intensities via area under the curve**					
	**experiment 1**		**experiment 2**		**experiment 3**	
	DAES	MBP apolipoprotein A1	DAES	MBP apolipoprotein A1	DAES	MBP apolipoprotein A1
0 h	10,969,711	8,924,104	14,380,752	13,124,388	12,194,024	12,817,510
1 h	10,223,146	8,544,660	3,645,761	2,178,447	8,371,075	11,369,610
3 h	6,218,589	6,959,711	5,149,761	7,222,711	2,963,104	7,792,560
6 h	3,622,740	5,479,882	4,575,175	9,388,004	1,818,497	2,742,983
24 h	4,216,296	6,210,882	5,055,711	11,892,782	2,321,569	5,132,711

Additional file 11 EGFR expression in HCT 116 and MDA-MB-453 cells

This file shows the Western blot that was used to examine the EGFR expression of HCT 116 and MDA-MB-453 cells.

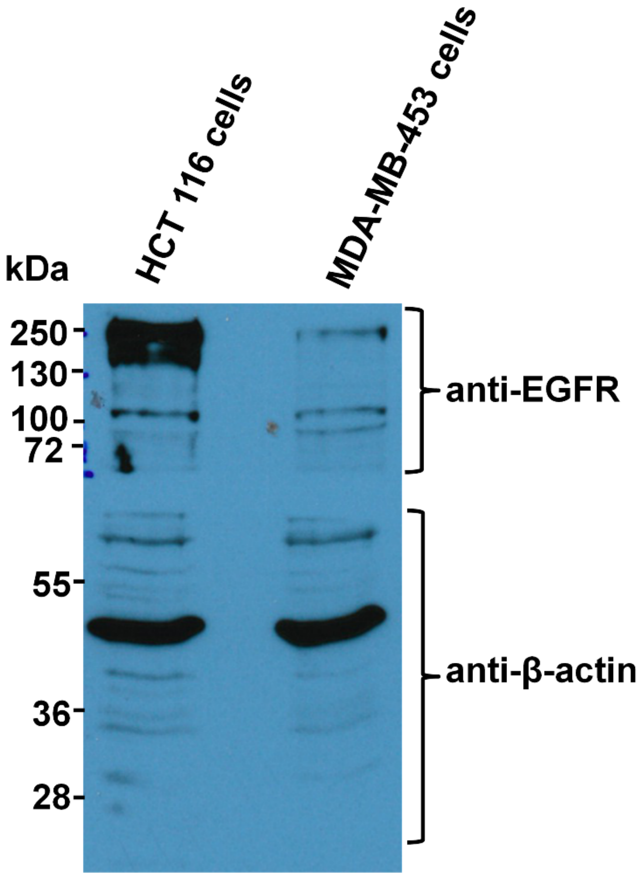

Additional fig. 27 EGFR expression in HCT 116 and MDA-MB-453 cells was analyzed by Western blotting of the cell lysates of HCT 116 and MDA-MB-453 cells. After blotting the SDS-PAGE gel, the membrane was divided into an upper and a lower half. Proteins in the upper part of the membrane were then detected by an anti-EGFR antibody, while an anti-β-actin antibody was used for the lower part. The EGFR has a molecular mass of about 180 kDa. β-actin (42 kDa) served as control. In anti-EGFR Western blot, an intense band within the HCT 116-lane was observed between 130 and 250 kDa, suggesting distinct expression of EGFR in HCT 116 cells. For MDA-MB-453 cells, a band with more than 10-fold lower intensity at the same molecular mass was noticed, indicating EGFR-expression in MDA-MB-453 cells at low levels. β-actin was detected in both cell lines to a similar extent.

Additional file 12 Cytotoxicity assays with NIH3T3 and HER14 cells

This file shows the results of cytotoxicity assays with DE and DAES with addition of SO1861 on the mouse fibroblast cell lines NIH3T3 and HER14.

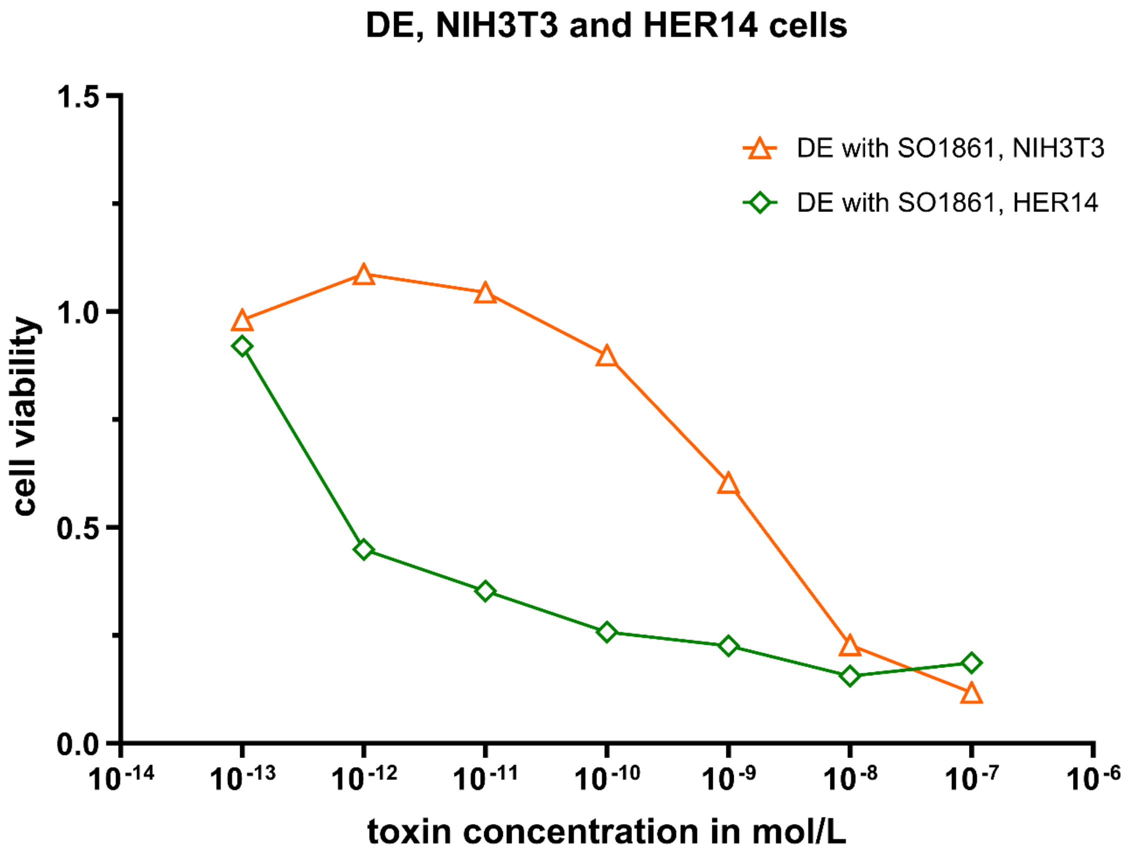

Additional fig. 28 Cytotoxicity assay, DE, NIH3T3 and HER14 cells

DE in combination with SO1861 was examined on NIH3T3 cells (mouse fibroblast cells, expressing no human EGFR) and HER14 cells (NIH3T3 cells transfected with human EGFR).

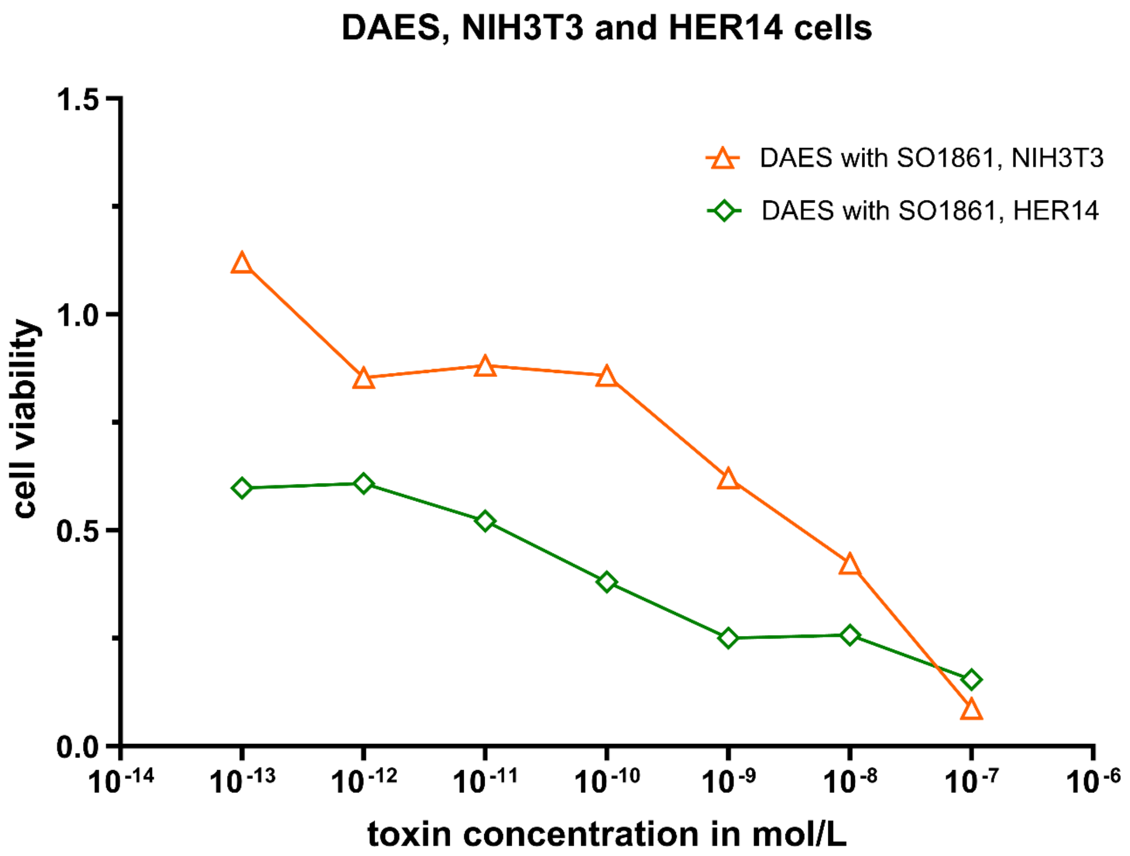

Additional fig. 29 Cytotoxicity assay, DAES, NIH3T3 and HER14 cells

DAES in combination with SO1861 was examined on NIH3T3 cells (mouse fibroblast cells, expressing no human EGFR) and HER14 cells (NIH3T3 cells transfected with human EGFR).
